# Genome-wide association of barley plant growth under drought stress using a nested association mapping population

**DOI:** 10.1186/s12870-019-1723-0

**Published:** 2019-04-11

**Authors:** Anh-Tung Pham, Andreas Maurer, Klaus Pillen, Chris Brien, Kate Dowling, Bettina Berger, Jason K. Eglinton, Timothy J. March

**Affiliations:** 1 School of Agriculture, Food and Wine, University of Adelaide, Waite Campus, Urrbrae, SA 5064 Australia; 2 Institute of Agricultural and Nutritional Sciences, Martin Luther University Halle-Wittenberg, Betty-Heimann-Str. 3, 06120 Halle, Germany; 3 Phenomics and Bioinformatics Research Centre, University of South Australia, North Terrace, Adelaide, SA 5000 Australia; 4 Australian Plant Phenomics Facility, The Plant Accelerator, University of Adelaide, Waite Campus, Urrbrae, SA 5064 Australia; 5 Sugar Research Australia, 71378 Bruce Highway, Gordonvale, QLD 4865 Australia

**Keywords:** Barley, Drought stress, Genome-wide association study (GWAS), HEB-25, Nested association mapping, QTL

## Abstract

**Background:**

Barley (*Hordeum vulgare* L.) is the fourth most important cereal crop worldwide. Barley production is compromised by many abiotic stresses including drought. Wild barley is a valuable source of alleles that can improve adaptation of cultivated barley to drought stress.

**Results:**

In the present study, a nested association mapping population named HEB-25, consisting of 1420 BC_1_S_3_ lines that were developed by crossing 25 different wild barley accessions to the elite barley cultivar ‘Barke’, was evaluated under both control and drought-stressed conditions in the Australian Plant Phenomics Facility, University of Adelaide. Overall, 14 traits reflecting the performance of individual plants in each treatment were calculated from non-destructive imaging over time and destructive end-of-experiment measurements. For each trait, best linear unbiased estimators (BLUEs) were calculated and used for genome-wide association study (GWAS) analysis. Among the quantitative trait loci (QTL) identified for the 14 traits, many co-localise with known inflorescence and developmental genes. We identified a QTL on chromosome 4H where, under drought and control conditions, wild barley alleles increased biomass by 10 and 17% respectively compared to the Barke allele.

**Conclusions:**

Across all traits, QTL which increased phenotypic values were identified, providing a wider range of genetic diversity for the improvement of drought tolerance in barley.

**Electronic supplementary material:**

The online version of this article (10.1186/s12870-019-1723-0) contains supplementary material, which is available to authorized users.

## Background

Barley (*Hordeum vulgare* L.) is the fourth most important cereal crop worldwide in terms of production and the second most important cereal crop in Australia [1]. It is used for multi-purposes such as food for animals and human, and further processed as malt for the food and beverage industry [2]. With the world population estimated to reach 9.1 billion in 2050, global cereal production will need to increase 35% from the current level of 2.1 billion tonnes per annum [3]. One limitation in achieving this production target is abiotic stress, particularly drought, which can result in large yield losses globally. Major drought events are forecasted to intensify due to global warming and uncertainties in rainfall patterns [4]. In Australia in 2002/03 and 2006/07 growing seasons, barley production was decreased by 55 and 56%, respectively due to severe drought [5, 6]. Due to the magnitude of the problem, the improvement of crop performance under drought conditions has become a global issue [7].

Understanding the genetic basis of drought tolerance in crop plants is useful for developing superior genotypes through conventional breeding. In the past, most studies have concentrated on water deficit during the late stages of barley development, in which post-harvest parameters were measured (i.e. yield and kernel weight) [8–11]. However, there have been accumulated reports in various cereal crops, including barley, that early growth stage parameters (e.g. tiller number, biomass formation, etc.) are highly correlated with yield potential and grain quality at harvest under both normal and drought conditions in the field [12–15]. In rice, broader leaves and rapid canopy growth were found to enhance the performance of plants exposed to drought stress [16, 17].

The advancement in digital imaging technology has enabled the performance of plants to be measured with higher precision [18, 19]. High throughput phenotyping technology was used by Honsdorf et al. [18] to study drought tolerance in wild barley introgression lines at the vegetative stage and identified a number of beneficial QTL, one of which improved biomass in the water deficit treatment by up to 35%. The high correspondence of the QTL found from this study with QTL previously identified in field trials for the same set of traits indicated that phenotyping juvenile plants using digital technology may assist in predicting adult plant performance.

It is common for genetic studies on drought tolerance in plants to find multiple QTL with small effects associated with the measured phenotypes, reconsolidating the previously known nature of drought as a multigenic trait with low-heritability and large genotype by environment (GxE) interactions [18, 20–22]. To improve the power of detecting QTL associated with complex traits exhibiting small-effect QTL, a novel mapping strategy was introduced entitled nested association mapping (NAM). NAM has the advantage of combining the high detection power of the linkage mapping method with the high resolution and greater allelic diversity of the association mapping strategy [23, 24]. NAM was first applied in maize and sorghum and was shown to have high power to detect QTL with small additive effects in a genome-wide approach for key traits such as flowering time, kernel composition, or disease resistance [25–28].

The domestication process has caused a genetic bottleneck in the elite germplasm of many crops including barley, which will limit future genetic gains in crop productivity, particularly in regard to newly emerging biotic or abiotic stresses [29–31]. Wild barley germplasm from the Fertile Crescent region has been identified as a source of germplasm with improved drought tolerance [32]. Drought tolerant barley varieties were developed using a wild barley line from Palestine, which produced 15% more grain yield than the control lines under dry-land growing conditions [33, 34].

The first barley NAM population, entitled HEB-25 [35], was created from crossing 25 genetically diverse wild barley accessions originating from the Fertile Crescent region to the malting cultivar ‘Barke’. This population consisted of 1420 individual BC_1_S_3_ lines and was genotyped with the barley Infinium iSelect 9 k chip consisting of 7864 *single nucleotide polymorphisms* (SNPs). GWAS using the HEB-25 NAM population has recently been demonstrated as an effective tool for gene identification in barley for traits such as flowering, salinity tolerance and plant development [35–39]. The HEB-25 population presents a reservoir of genetic diversity that can be exploited for variety development.

The aims of the present study were to i) evaluate the growth response of the HEB-25 population grown under drought stressed conditions during early developmental ii) identify QTL from wild barley that can improve plant growth under drought stressed conditions and identify candidate genes underlying those QTL.

## Results

### The dynamics of shoot area, absolute and relative growth rate during the course of the experiment

**Fig. 1:**
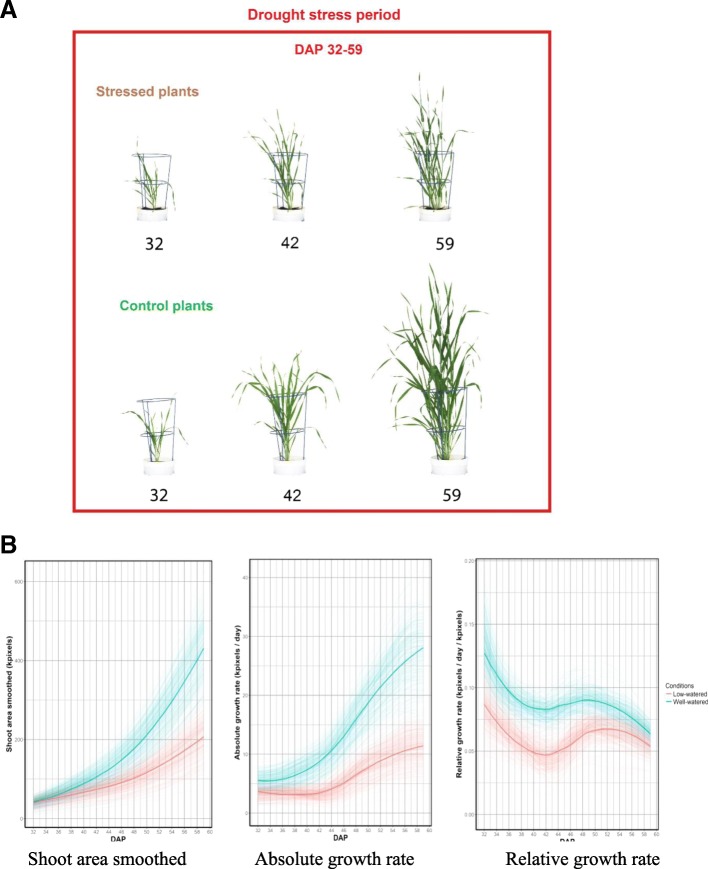
Monitoring the dynamics of plant growth throughout the experiment. **a** Plants were non-destructively imaged from 32 to 59 days after planting under drought and non-stressed treatments. **b** Plots for shoot area smoothened (SAsm), absolute growth rate (AGR) and relative growth rate (RGR) under control (cyan) and drought (red) treatment in the north east Smarthouse at the Plant Accelerator, University of Adelaide in the year 2014 are shown as an example. The bold line represents the average of each treatment

Across three consective years, the HEB-25 population was evaluated under water-limited and control conditions that were applied at 32 days after planting (DAP) until the completion of the experiment at 59 DAP (Fig. 1a). Plots for Shoot area smoothened (SAsm), Absolute growth rate (AGR), and Relative growth rate (RGR) across the 3 years are shown in Additional file 1: Figure S1 and for 2014 in the North East (NE) Smarthouse, as an example, is shown in Fig. 1b. Three intervals with distinct kinetics were observed for AGR and RGR including 32–40 DAP, 42–50 DAP, and 52–59 DAP. The AGR and RGR were calculated for three intervals that captured these three phases.

The RGR plot for 2014 was different from those of 2015 and 2016. In 2014, RGR interval from 42 to 50 DAP showed an increasing trend, while it was decreasing for 2015 and 2016. To investigate this, climatic data within each Smarthouse was examined and compared across the 3 years. No association with either min, max temperature or growing degree days was observed with the difference in RGR (Additional file 2: Figure S2). The macro climatic conditions in the two Smarthouses thus appeared to be comparable across the years. Therefore the difference observed in RGR in the interval from 42 to 50 DAP in 2014 could not be explained.

### Effect of treatment, genotype and experiment setting on phenotypic variation

Treatment was found to be significant in all 3 years and for all traits, but the genotype effect was trait-dependant (Additional file 3: Table S3). In 2014, seven traits showed no significant genotype effect, including SAsm, AGR32, RGR42, RGR52, DW, FW, and TN. In 2015, all traits had a significant genotype effect. In 2016, RGR32 and WUE had no significant genotype effect.

Genotype x treatment interaction was found to be significant for 3, 11 and 6 traits in 2014, 2015, and 2016, respectively. Two traits, which did not have a significant genotype x treatment effect in any of the years, were WUE and tiller number.

There was a significant effect of the Smarthouse as well as the interaction between Smarthouse x position for all 14 traits investigated across 3 years. Trait value means of plants grown in the north-east Smarthouse were generally higher compared to those in the north-west Smarthouse (data not shown). One possible explanation was that the north-east Smarthouse receives more light, especially in the morning, which resulted in increased plant growth.

Plant growth stage was used as a covariate in modelling. Most of the lines were at growth Zadoks stage 33, with a few lines reaching Zadoks stage 49 and above. In general growth stage was not significant and dropped out of the model for most of the traits (Additional file 3: Table S3).

### Trait performance

Means of the traits SAsm, dry weight (DW), and fresh weight (FW) in the control treatment were 2–2.5 times higher than means of these traits in the drought treatment across the 3 years (Additional file 4: Table S4). The only exception was WUE where the mean values under control treatment were 55–67% of the means of the drought treatment.

Heritability of each trait was generally higher for control treatment compared to the drought treatment ranging from 0.27 to 0.85 in control treatment and 0.34 to 0.80 in the drought treatment across the 3 years. HEI had the highest heritability in both treatments in all 3 years. RGR42, WUE, and RGR32 had the lowest heritability for the year 2014, 2015, and 2016, respectively.

To compare the growth response under drought treatment of genotypes with such large variation in plant size and growth, we calculated the ratio of the phenotypic values between SAsm, DW, HEI, TN in drought stress versus the control treatment. Ratios represent the drought response independent of plant size/growth parameters in control conditions. Ratios that are close to 1 indicate a high capacity to maintain the four parameters, whereas ratios that are smaller than 1 indicate a larger reduction. For all of four selected traits there was no significant correlation between the ratio of SAsm, DW, HEI and TN in drought stress versus control and the corresponding phenotypic values in control treatment (*r* < 0.2 for DW and SAsm, and *r* < 0.08 in all years for both TN and HEI) (Additional file 5: Figure S6A, B, C and D).

### GWAS results

#### Dry weight (DW)

**Fig. 2:**
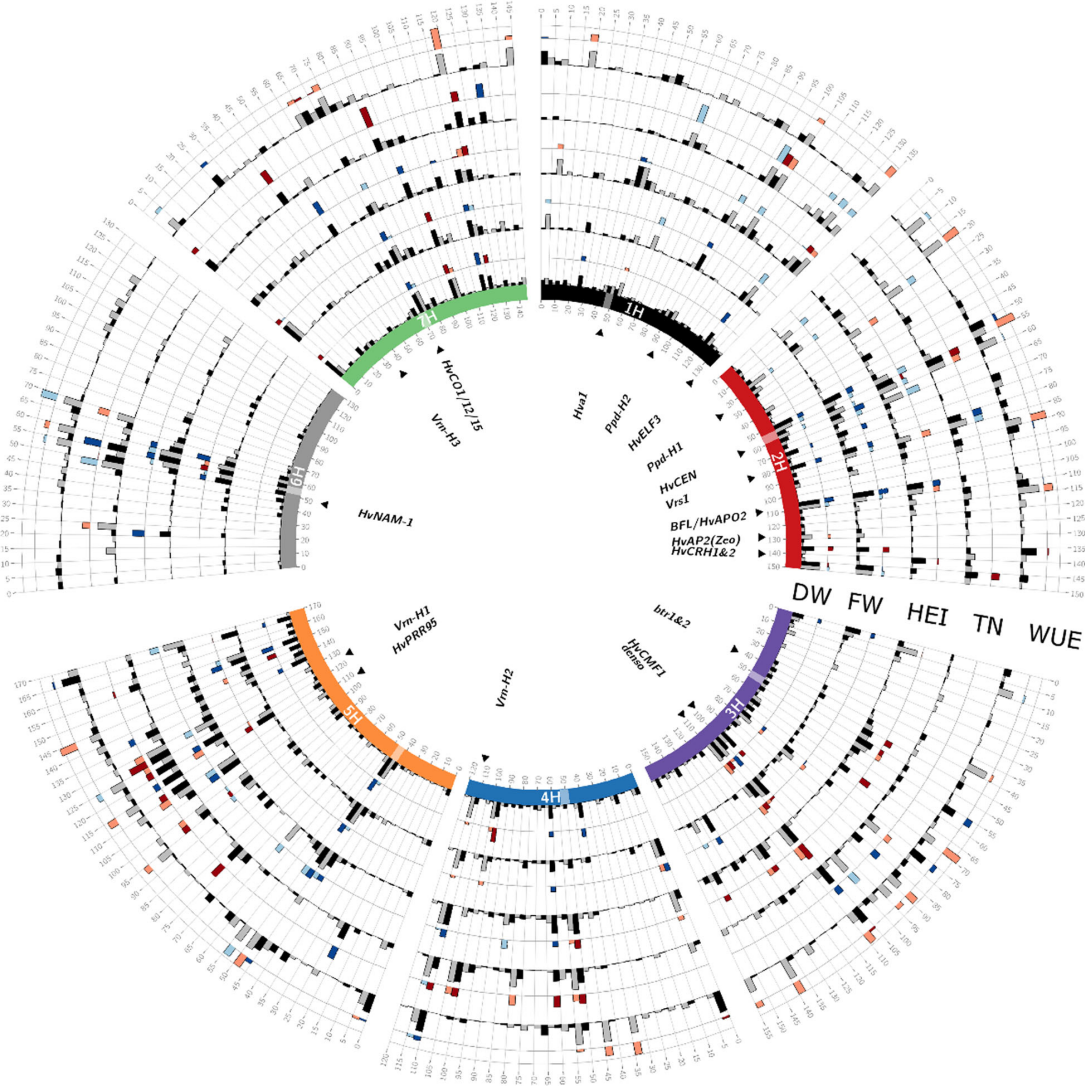
Comparison of GWAS results across five of the post-harvest destructively measured traits. The data in this Circos plot results from 100 cross-validated (20 times 5-fold) GWAS runs performed within each treatment for the five studied traits including dry weight (DW), fresh weight (DW), plant height (HEI), tiller number (TN), and water use efficiency (WUE). Barley chromosomes are shown on the inner circle with different colors and centromeres are indicated with transparent boxes. For each trait, the first (inner) track represents the frequency of QTL detection in a 5-cM window while the outer track represents the effect of this QTL. The maximum height of the effect bars for each trait are 1.3 g for DW, 9 cm for HEI, 1.82 for TN, 0.1 g/g water for WUE. Window positions (in cM, following Maurer et al. 2015) are ordered clockwise per chromosome. In the inner track, QTL appearing under control and drought stress treatment are represented with black and gray bars, respectively. The effect of the QTL conferred by the wild allele relative to Barke is represented on the outer track, where blue and red bars indicate decreasing and increasing wild barley QTL effects, respectively for each treatment. Candidate genes, potentially explaining the observed QTL effects, are indicated inside the inner circle

There were 26 QTL detected in the control treatment that explained 44% of the total phenotypic variance (Vp). In the drought treatment, 24 QTL were identified and accounted for 37% of Vp. Out of the 39 QTL for DW, 11 were detected in both treatments and 17 were treatment-specific (Fig. 2 and Additional file 6: Table S6). The QTL that explained the most phenotypic variance for DW in control and drought treatment was QDw.HEB25-7H.2 at 70.4 cM (6%) and QDw.HEB25-3H.4 at 108 cM (5%), respectively. At the common QTL QDw.HEB25-4H.4 (97.2 cM), wild alleles had a mixed effect (both increasing and decreasing) in both treatments, with the allele from family F01 increasing DW the most by up to 1.32 g (equivalent to 17% increase) in the control treatment. In the drought treatment, wild alleles from 20 out of 25 families at this QTL increased dry weight compared to the Barke allele, with those from family F01 and F07 increased DW up to 0.3 g (approximately 10% increase).

#### Fresh weight (FW)

Twenty-nine QTL were detected in the control treatment, which explained a total of 42% Vp and 25 QTL were detected in the drought treatment, which explained 39% of Vp (Additional file 6: Table S6). The QTL that explained the most Vp for drought stress and control treatment were QFw.HEB25-2H.4 at 64.4 cM (4.5%) and QFw.HEB25-7H.2 at 67.8 cM (5%), respectively. Due to the high correlation between DW and FW (Additional file 7: Figure S5), 20 out of 24 QTL detected for DW in drought treatment were also detected for FW in drought treatment, and 19 out of 26 QTL detected for DW in control treatment were also detected for FW in control treatment. In the drought treatment, wild alleles at QFw.HEB25-3H.5 (107.05 cM) wild alleles increased FW the most (up to 9.8 g, equivalent to 37% increase in FW). In control treatment, at the QTL QFw.HEB25-3H.6 (117 cM), wild alleles increased FW the most (up to 12.0 g in family F08 or 10.5% increase in FW compared to the Barke alleles).

#### Tiller number (TN)

There were 18 loci detected for tiller number in the control treatment which explained 41% of Vp (Fig. 2 and Additional file 6: Table S6). In the drought treatment, 21 loci were detected and explained 46% of Vp. Among the total 39 QTL detected for TN, 7 were common between two treatments. The QTL QTn.HEB25-5H.5 which co-localized with the vernalisation gene *VRN-H1* explained the highest phenotypic variation (up to 10% in the control and 14% in drought treatment). At the *VRN-H1* locus, wild alleles from all families increased tiller number up to 6.0 in the control treatment and up to 3.3 in the drought treatment. Among the 13 drought treatment specific QTL, wild alleles at two QTL that co-localized with *HvELF3* and *Ppd-H1* decreased tiller number in all families except for family F23.

#### Plant height (HEI)

There were 23 and 31 SNPs detected for HEI in the drought and control treatment, respectively, of which 16 were common across treatments (Additional file 6: Table S6). Among 38 loci were identified for HEI, 23 locating near known genes controlling flowering and plant architecture in barley. The Vp explained by all QTL for plant height in both control and drought treatment was 60%. The most significant association for plant height was observed at the QTL QHei.HEB25-3H.5 (108 cM) explaining 22 and 11% of the variance in the control and drought treatment, respectively. This locus co-localized with the semi-dwarfing *sdw1*/*denso* gene in barley. Other major QTL that explained from 5 to 10% of phenotypic variation include QHei.HEB25-3H.6 (122 cM), QPh.HEB25-5H.3 (107.5 cM), and QHei.HEB25-5H.4 (122.6–125 cM). The effect of the wild alleles at the *sdw1/denso* locus in each family within the drought treatment is in general half of that in the control treatment. Wild alleles from 25 HEB families at *sdw1/denso* locus increased plant height up to 8.9 cm in control and 5.9 cm in drought treatment. In contrast to *sdw1/denso,* at five other common QTL including QHei.HEB25-3H.4 (113.4 cM), QHei.HEB25-5H.1 (41.6–45.2 cM), QHei.HEB25-5H.3 (107 cM), QHei.HEB25-5H.4 (125 cM), and QHei.HEB25-6H.2 (52.1–55 cM), the wild alleles from most families reduced plant height.

#### Shoot area smoothed (SAsm)

**Fig. 3:**
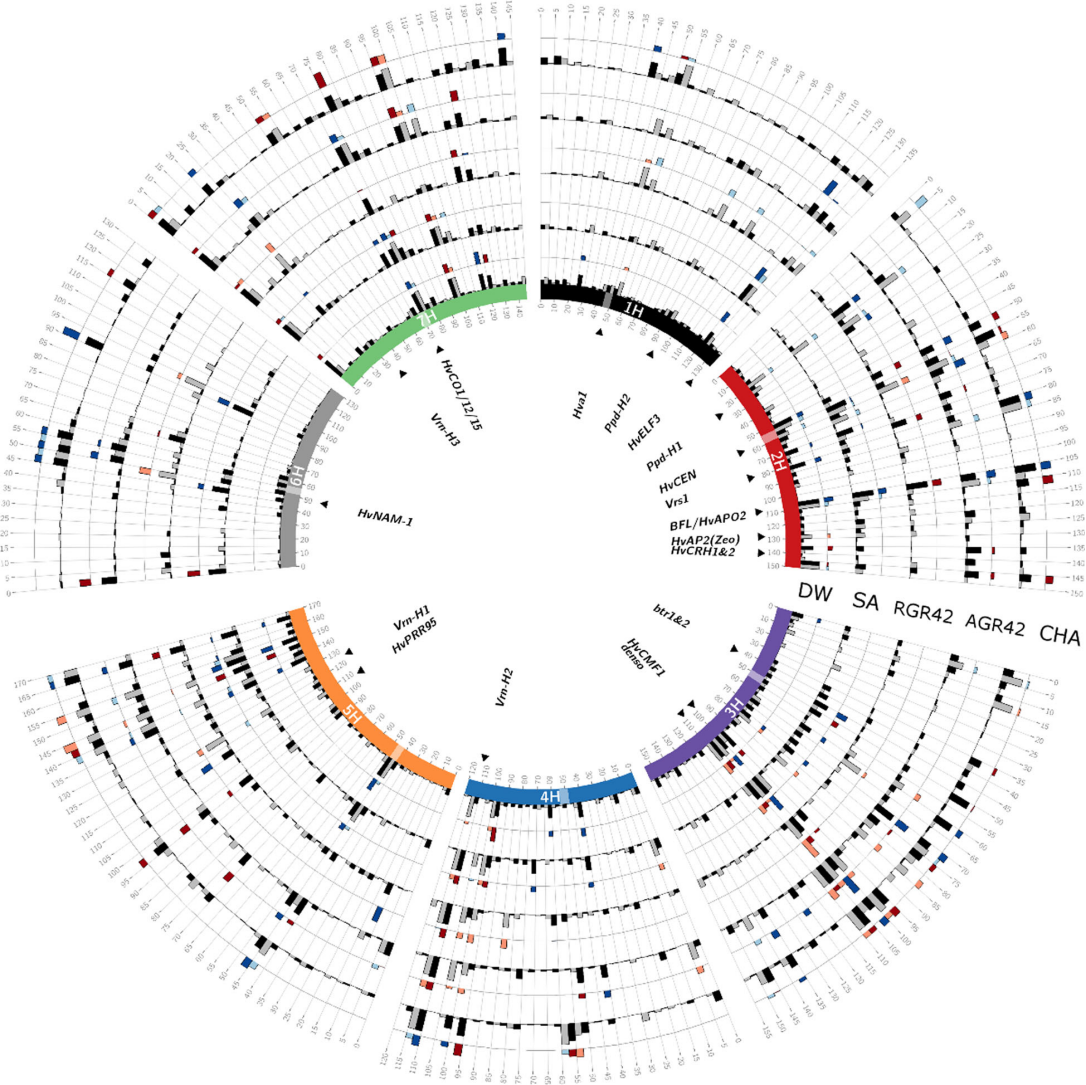
Comparison of GWAS results of dry weight relative to four non-destructive imaging determined traits. The data in this Circos plot results from 100 cross-validated (20 times 5-fold) GWAS runs performed within each treatment for the five studied traits including dry weight (DW), shoot area smoothed (SAsm), absolute growth rate 42–50 dap (AGR42), relative growth rate 42–50 dap (RGR42), and convex hull area (CHA). Barley chromosomes are shown on the inner circle with different colors and centromeres are indicated with transparent boxes. For each trait, the first (inner) track represents the frequency of QTL detection in a 5-cM window while the outer track represents the effect of this QTL. The maximum height of the effect bars for each trait are 1.3 g for DW, 98.6 kpixels for SA, 4.44 kpixels/day for AGR42, 0.0039 kpixels/day/kpixels for RGR42, 654 kpixels for CHA. Window positions (in cM, following Maurer et al.2015) are ordered clockwise per chromosome. In the inner track, QTL appearing under control and drought stress conditions are represented with black and gray bars, respectively. The effect of the QTL conferred by the wild allele relative to Barke is represented on the outer track, where blue and red bars indicate decreasing and increasing wild barley QTL effects, respectively for each treatment. Candidate genes, potentially explaining the observed QTL effects, are indicated inside the inner circle

There were 27 and 34 QTL detected under the drought stress and control treatment, respectively (Fig. 3 and Additional file 6: Table S6). Sixteen QTL were found to be common between the two treatments. Six out of 15 common QTL for SAsm were also detected as the common QTL for dry weight. The effect of the wild alleles at common QTL between SAsm and DW were also very similar. The QTL that explained the most phenotypic variance for SAsm in both the control and drought treatment was QSasm.HEB25-3H.7 (6%). At the QTL QSasm.HEB25-3H.7 locating near *HvCMF1* gene, wild alleles increased SAsm in most of the families across both treatments. At all other QTL, the effect of wild alleles for SAsm was mixed across QTL and families.

#### Absolute growth rate (AGR)

There were five common genomic regions detected in the drought treatment across all three AGR intervals, including 1H (48.5–48.9 cM), 1H (128.3–130.35 cM), 2H (106.8–109.3 cM), 3H (100.4–104.8 cM), 7H (99.8–102.2 cM) and 55 interval-specific QTL. Among the common QTL, the wild alleles at 3H (100.4–104.8 cM) increased AGR in most families. Within the control treatment, there were 11 QTL common across all three AGR intervals and 52 interval-specific QTL. Among the 11 common QTL, six located near known genes including *Ppd-H1*, *BFL/HvAPO2, VRN-H2, HvCO15, HvCCA1/HvLHY, HvCMF1,* and *HvCO6.* The genomic regions on chromosome 2H from 106.8 to 109.6 cM and 3H from 100.4 to 106.1 cM were detected across all three AGR intervals in both treatments. In control treatment, the QTL where the wild alleles showed the largest positive effect was on chromosome 6H at 5.6 cM for all three intervals. In drought stress, QTL with the largest effect for AGR32, AGR42 and AGR52 were QAgr32.HEB25-3H.4 at 100.4 cM, QAgr42.HEB25-3H.6 at 103.8 cM, and QAgr52.HEB25-3H.5 at 104.8 cM, respectively. As AGR42 was highly correlated with the other two intervals (Additional file 7: Figure S5), details for QTL detected for this interval are summarised below.

For AGR42, there were 26 and 29 QTL detected for drought stress and control treatment, respectively (Additional file 6: Table S6). There were 15 common QTL between two treatments, ten of which reside near known flowering genes. The QTL that explained the highest Vp (up to 6%) in the drought stress and control treatment was QAgr42.HEB25-3H.6. In the drought treatment, the wild alleles at QTL QAgr42.HEB25-3H.3 (55.5 cM), QAgr42.HEB25-3H.6 (103.8 cM), and QAgr42.HEB25-4H.4 (103.9 cM) showed the highest trait-increasing effects. In the control treatment, several QTL with beneficial wild alleles that increased AGR42 were detected such as QAgr42.HEB25-2H.6 (146.4 cM), QAgr42.HEB25-3H.5 (89.1 cM), QAgr42.HEB25-3H.6 (106.1 cM), QAgr42.HEB25-6H.1 (5.6 cM), and QAgr42.HEB25-7H.6 (120 cM).

#### Relative growth rate (RGR)

There were 22, 25 and 23 QTL detected for RGR32, RGR42, and RGR52 in the drought treatment, respectively. There were six genomic regions detected across all three intervals including 1H (126–128 cM), 2H (18.9–23 cM), 2H (55.6–62 cM), 3H (131–135.5 cM), 4H (99.6–101.4 cM), and 7H (23–29.6 cM). Four out of these QTL located near known developmental genes including *HvELF3*, *Ppd-H1*, *HvCEN*, and *HvCMF4*. Similarly, there were 19, 24, and 21 QTL detected for RGR32, RGR42, and RGR52 in the control treatment, with three common QTL found across all intervals including 2H (18–26 cM), 4H (111.3 cM), and 5H (14.5 cM). Across both treatments there were 7, 10 and 6 common QTL for RGR32, RGR42, and RGR52, respectively. At one of these common genomic regions, 2H (18.9–23 cM), which is in close proximity to *Ppd-H1*, the wild barley alleles from all families reduced RGR in the drought treatment for all three intervals, while the effect was mixed depending on families and RGR interval in the control treatment. As RGR42 was highly correlated with the other two intervals, details for QTL detected for this interval are summarised below.

For RGR42, there were 25 and 24 QTL detected under drought stress and control treatment, respectively, with ten QTL shared between the two treatments (Additional file 6: Table S6). Among the treatment-common QTL for RGR42, four were also detected for DW including QRgr42.HEB25-2H.2, QRgr42.HEB25-2H.6 (141–149 cM), QRgr42.HEB25-7H.1 (0.2–2.5 cM) and QRgr42.HEB25-7H.4 (97.2–100.25 cM). Other common QTL for RGR42 were residing near known flowering genes including *Ppd-H1*, *Ppd-H2*, *HvCMF1*. At all common QTL, the wild alleles had a mixed effect across the families.

#### Convex hull area (CHA)

There were 27 and 28 QTL detected in the drought stress and control treatment, respectively (Fig.3 and Additional file 6: Table S6). There were 14 common QTL between the two treatments, and four of these were also detected for DW (the trait that CHA most correlated with). The QTL QCha.HEB25-3H.7, which located near the gene *sdw1/denso*, explained the highest Vp (10%) in both treatments. Similar to the effect of *sdw1/denso* on plant height, the wild alleles from all families at this locus increased CHA in both treatments. There were two additional QTL where the wild alleles from most of the 25 families increased CHA in both treatments including QCha.HEB25-5H.6 (152 cM), and QCha.HEB25-7H.3 (51 cM). The alleles from family F14 increased CHA the most at all of these three QTL.

#### Caliper length (CL)

Due to the high correlation between CHA and CL, the GWAS results for CHA and CL were similar. In the control treatment, 23 QTL were detected for CL and 19 of them were also found for CHA. In the drought treatment, 22 QTL were identified and 15 of them were shared between CL and CHA (Additional file 6: Table S6).

#### Water use efficiency (WUE)

There were 33 and 22 QTL detected in the drought stress and control treatment, respectively, including 10 common QTL detected across both treatments (Additional file 6: Table S6). The QTL that explained the most phenotypic variance was QWue.HEB25-2H.6 at 139.9 cM (3.5%) in the control treatment and QWue.HEB25-3H.10 at 154.8 cM (7%) in the drought stress treatment. In the drought stress treatment, wild alleles at QTL QWue.HEB25-3H.6 (87.4 cM) increased WUE in all families and wild alleles at QTL QWue.HEB25-6H.2 (37 cM) reduced WUE in all families, all other QTL showed a mixed effect for the wild alleles. At QTL QWue.HEB25-7H.6 (116.1 cM), the wild alleles from family F02 increased WUE the most compared to the Barke allele (10.8% increase).

#### QTL associated with multiple traits

When all significant QTL identified for the traits DW, HEI, TN, SAsm, CHA, WUE, AGR42 and RGR42 were compiled, QTL associated with multiple traits were identified. When QTL within a 4 cM window were grouped into a single QTL, which is similar to the criteria set by Maurer et al. [36], 21 genomic regions were found to be associated with at least 4 traits or more. The genomic region on chromosome 2H co-localizing with the gene *HvCEN* was found to associate with all of the traits, six of which were detected in both treatments including DW, TN, HEI, AGR42, RGR42, and WUE. The second most common genomic regions (associated with seven traits, excluding TN) were on chromosome 2H at 109 cM and on chromosome 4H at 113 cM and these two co-localized with *BFL (BARLEY FLORICAULA/LEAFY)/HvAPO2* and *VRN-H2* genes, respectively. Regions that were associated with six different traits were 3H (105–108 cM), 4H (97–104 cM), 5H (0–3.8 cM), 5H (144.2–149.8 cM), 5H (165.8–169.4 cM), 7H (0.2–2.5 cM), and 7H (70.2–72.5 cM).

## Discussion

### The effect of the drought treatment on the HEB-25 population

This study aimed to evaluate the response of the HEB-25 population when grown under control and drought treatments. Plant growth in both treatments was measured in a non-destructive manner using a high-throughput imaging system.

The reduction in SAsm due to drought stress was observed 5–8 days after the drought treatment was commenced (depending on the genotype). By 59 DAP the drought treatment reduced SAsm by approximately 50% compared to the control. This is in accordance with previous observations that it takes 6–7 days until differences in growth between control and drought stressed plants are observable [40, 41]. In contrast, the difference in AGR and RGR between the two treatments was evident at the onset of the drought stress, suggesting an immediate effect. This is expected as it has been well demonstrated that leaf growth, determined by cell division and expansion, is highly sensitive to water stress and can be reduced by 50% within 24 h after the stress is induced [42–45].

It has been reported that small plants or plants with smaller leaves tend to have better drought tolerance because they transpire less water [46, 47]. In contrast, in our study, growth reduction under drought was independent of the plant size under normal conditions. There was a small or no correlation between the ratio of shoot area, dry weight, tiller number, and plant height in drought stress versus control and the corresponding phenotypic values in control treatment. This lack of correlation between plant size and drought tolerance was also observed in *Arabidopsis* [48], which showed that larger plants in normal conditions were able to maintain both stress tolerance and improved growth when experiencing drought.

### Time-dependent QTL detected for barley plant growth

The GWAS analysis revealed that there are few common QTL detected across the three intervals measured for the growth rate-related traits. For AGR, there were only two QTL shared among three intervals in both treatments. For RGR, only one common QTL, co-localizing with *Ppd-H1,* was found for three intervals in both treatments. There were more common QTL found between two intervals (i.e. seven QTL were common for AGR42 and AGR52), which reflected the high correlation between these two. Beside the common QTL, interval-specific QTL were found for three intervals indicating different gene action and interactions occurred within each interval. For example, for RGR in the control treatment, besides the three QTL common across all intervals, loci co-segregating with known genes such as *HvELF3,* and *Vrn-H2* were among those identified for the first interval, *Ppd-H2*, *HvCEN*, *Zeo, HvPRR95* were found for the second, and *Vrn-H1 and HvCMF1* were detected for the third interval. Time-dependent QTL mapping for plant growth was also reported in *Arabidopsis* [49, 50], in rice [51], in *Setaria* [52], and maize [53]. To our knowledge, this is the first study in barley that has mapped time dependent QTL for the absolute and relative growth rate of barley seedlings. The large distinction of the QTL detected for each interval suggests that measuring growth for the different intervals is to be favoured over single interval measurements.

As mentioned above, most of the QTL detected for AGR and RGR co-localized with developmental genes such as *HvCO1/2/3/6, HvCMF1/4/7, Ppd-H1/2*, *VRN-H1/2*, *HvCEN,* and *HvELF3*. Elberse et al. [54] detected a QTL on chromosome 6H (11 cM) for both relative growth rate and seed mass, which was also detected in our study for RGR42. Yin et al. [55] reported a weak association of *sdw1/denso* with relative growth rate. In our study, the genomic region on chromosome 3H from 100 to 104 cM was detected for AGR and RGR in all of three intervals, but this could be either *sdw1/denso* or *HvCMF1*. Poorter et al. [56] and Van Rijn et al. [57] reported three major QTL for RGR on 1H, 2H, 5H and one minor QTL in 6H. Honsdorf et al. [18] detected three QTL for AGR on 3H, 4H and 6H and no QTL for RGR. The QTL QAgr42.HEB25-3H.7 (143.5 cM) and QAgr52.HEB25-3H.6 (139 cM) detected in our study seem to be similar to the QTL on chromosome 3H reported by Honsdorf et al. [18].

### QTL detected for traits measured at harvest

QTL located on all seven chromosomes were detected for DW, FW, TN, and HEI measured in this study, confirming the quantitative nature of these traits.

For plant dry biomass, our study identified 11 common and 28 treatment-specific QTL. Six of the 15 QTL identified in our study for drought stress on 2H, 3H, 4H, 5H and 7H were within 5 cM of those reported by Wehner et al. [21]. Honsdorf et al. [18] described four QTL for DW on 3H, 4H and 6H, with the 4H QTL likely to be coincidental with the QTL QDw.HEB25-4H.4 found in this study. Von Korff et al. [58] reported six QTL on chromosome 2H-5H and 7H for dry biomass in a wild barley advanced backcross population of which three QTL on 2H, 3H and 4H coincided with QTL detected in this study. The QTL on chromosome 4H-97.2 cM was reported across at least four different mapping studies, indicating its important role in biomass production in barley.

For plant height, although 70% of QTL identified for drought stress were also detected in control treatment, there were still distinctive QTL revealing different gene interaction for different environmental conditions. For example, QTL co-localizing with *Vrs1, Vrn-H3*, and *DWAFT 2* were identified in drought treatment while those locating near *HvCMF7*, *HvCO6*, *DWAFT5/14* were found in control treatment. Minor effect QTL have been reported throughout the literature for plant height by many groups [18, 58–62]. Despite the difference in minor genes, key genes controlling plant height including *sdw1/denso* and *sdw3* have been detected across all of these studies mentioned above. The *GA-20 oxidase* gene was suggested to be a candidate for the *sdw1/denso* locus [63]. The effect of wild alleles at these two QTL on reducing plant height was also reported by Honsdorf et al. [18] and von Korff et al. [58]. There are at least three novel loci detected for plant height in this study that have not been reported before. These three QTL were detected at high confidence level (greater than 90 out of 100 cross validations) and were located in close proximity to known genes including QPh.HEB25-5H.3 (near *Vrs2*), QPh.HEB25-5H.4 (near *VRN-1*) and QPh.HEB25-7H.5 (near *HvCMF7*).

Tiller number is an important yield-determining trait [64]. For tiller number, there were 18 QTL detected in the control treatment, 15 are in close proximity with QTL for tiller number reported by Alqudah et al. [59]. However, although *Ppd-H1* and *Vrs1* were identified as key genes regulating tiller number in spring cultivated and landrace barley by Alqudah et al. [59], only *Vrs1* was detected in both treatments whereas *Ppd-H1* was only detected under the drought treatment in our study. Using an AB-NAM population created by crossing 25 wild barleys selected from the wild barley diversity collection with cultivar Rasmusson, Nice et al. [65] identified *Ppd-H1* and a second QTL at 4H-91.29 cM (anticipated to be the *SUCROSE TRANSPORTER 1-HvSUT1*) associated with controlling productive tiller number in field conditions. In our study, four out of eight flowering genes including *HvCEN, sdw1/denso, VRN-H1, Ppd-H1* reported by Maurer et al. [35] were found to be associated with tiller number, together with other flowering genes such as *HvCO5/6/8/16*. QTL residing near *HEXOKINASE 2* and *3* genes and *HvSUT1* were also identified in our study in the control treatment*.* Recently, several studies highlighted the importance of sugars as a key component of plant branching [66, 67]. The finding from our study supports the view that flowering and sugar-related genes have a role in barley tillering. Novel QTL detected in this study for tiller number include QTn.HEB25-2H.4 (146 cM), QTn.HEB25-5H.1 (23.2 cM) and QTn.HEB25-5H.3 (77 cM).

### Candidate genes for common QTL detected for 14 traits

Many of the common QTL detected for 14 traits across both treatments co-localized with known developmental flowering regulator genes, such as *Ppd-H1, HvCEN*, *VRN-H1*, *VRN-H2, and sdw1/denso*, demonstrating the importance of these genes in barley development. Among these genes, the QTL residing in the close proximity with *HvCEN was* associated with all of the traits investigated in this study. *HvCEN* is a modifier of seasonal flowering response, with a missense mutation in the HvCEN protein (Ala135 to Pro) differentiating spring from winter barley cultivars [68]. In this study, the wild alleles at the *HvCEN* locus from 25 families generally reduced the phenotypic values for all of the traits, except for CHA, WUE, and TN. When the HEB-25 population was evaluated with salt stress in field conditions, wild alleles at the *HvCEN* locus also reduced plant height and dry mass per m^2^ but increased yield under stress and control treatments [37]. The authors indicated that the yield improvement effect of the wild *HvCEN* alleles is derived from an increased number of ears and from larger grains. *HvCEN* showed a same fashion of effect to the traits in this experiment compared to the salt tolerance study, indicating that this QTL potentially may have the capacity to enhance yield in both control and drought-stressed condition. However, we cannot completely rule out the possibility that the observed effect has been derived from another unknown gene, which is in linkage disequilibrium with *HvCEN*.

Another chromosome region at 1H-48 cM was associated with four traits (DW, HEI, AGR42, RGR42) in both treatments and is in close distance to *HvCMF10*, *HvHXK1*, and *Hva1*. Among the three candidate genes at 1H-48 cM, the barley *Hva1* gene is the best studied gene. It encodes a late embryogenesis abundant (LEA) protein and is well-known to enhance tolerance to drought in barley. Transformations of the barley gene into wheat, oat, rye, and mulberry all resulted in an enhanced tolerance to drought and salinity stress [69–72]. Little is known about the *HvHXK1* function in barley apart from its high transcript levels at night and its role in sugar signalling and targeting of carbon into downstream metabolic pathways [73, 74]. Similarly, the specific role of *HvCMF10* is unclear except its involvement in the control of flowering time [75]. As these three candidate genes are closely linked, we are currently unable to specify, which of those is the causative gene. Further fine mapping studies may reveal the importance of these genes for plant development and drought stress tolerance in barley.

## Conclusions

The ultimate goal of this study was to identify beneficial alleles from wild barley that can be used for improving drought tolerance in barley. However, it was demonstrated that any trait selected for drought tolerance has benefits as well as risks. Considering stress severity and the phase when drought stress typically occurs during plant development in a target environment are, thus, critical to breed for improved drought tolerance [76]. In this study, loci where alleles from wild barley that both increase and decrease phenotypic values under drought stress and control treatment were detected, providing a pool of usable alleles for breeding.

## Materials and methods

### Plant materials

The HEB-25 NAM population consists of 1420 BC_1_S_3_ lines derived from backcrossing 25 diverse wild barley lines (*Hordeum vulgare* ssp. *spontaneum* and *agriocrithon*) to the cultivar Barke. The resulting population comprises 25 sub-families each consisting of between 23 to 61 individual. The population construction was previously reported by Maurer et al. [35]. The University of Adelaide obtained this population from the Martin Luther University of Halle-Wittenburg (MLU) under a Material Transfer Agreement (MTA) no. A135366. The whole population subsequently underwent quarantine inspection following the regulations applied to imported plant research materials set by the Department of Agriculture and Water Resources of Australia.

### Experimental design

A total of 1343 HEB-25 lines were tested in drought stress experiment over 3 years, with 447 lines screened in 2014 and 448 lines each in 2015 and 2016. The first set contain lines from 9 families (HEB-03, 04, 09, 12, 13, 18, 20, 21, and 22), the second contain lines from 8 families (HEB-02, 05, 07, 11, 15, 16, 19, and 23) and the third set have lines from 8 remaining families (HEB-01, 06, 08, 10, 14, 17, 24, 25). The reason of using only a third of the NAM population was because there was a limitation in space in the Plant Accelerator so only one third of the NAM population could be screened for drought stress at a time. The families selected for each year were chosen so that the number of lines screened each year was approximately equal. The three experiments were executed at the same time each year, from 16th June (potting) to 16th August (last day of imaging). The experiment was accommodated in two automated greenhouses, so-called Smarthouses (North West (NW) and North East (NE)), at the Plant Accelerator greenhouse facilities in Adelaide, Australia (34°58′16.18″S; 138°38′23.88″E). Each Smarthouse was divided into six zones each comprising a grid of four lanes by 22 positions, as sets of four lanes were found to be homogeneous in terms of plant growth variability [77].

The design employed for each Smarthouse experiment was a split-plot design in which two consecutive carts form the main plot. The main-plot design was an unreplicated design with replicated check and recipient lines, which were Navigator and Barke, respectively. In order to deal with the anticipated spatial variation, lines were allocated to main plots using a blocked, row-and-column design, the blocks being the zones. A feature of the main-plot design was that, for the check lines, (i) there were 6 main plots in each zone, and (ii) there were 3 or 4 main plots in each column; the Barke main plots were similarly distributed across zones and columns. The subplot design merely randomized treatments (control, drought stress) to the two carts in each main plot.

At each position, there was a cart containing a pot with a single plant. Lines were allocated to the 24 × 11 = 264 pairs per Smarthouse. Of the 264 pairs available in a Smarthouse, 36 had Navigator, a control line, allocated to them, 224 (or 223 in the NW Smarthouse) had 224 (or 223) lines allocated to them and the remainders (4 or 5) had the recipient line, Barke, assigned to them. In general, Navigator was replicated 72 times and Barke 8–10 times per treatment while HEB lines were unreplicated. The main plot design was generated using the software DiGGer [78] and the subplot randomization was done using the R package dae [79]. More detailed information and the graphical layout of the experiment was listed in the Additional file 8.

### Plant growth conditions

The drought stress experiment was similar to that reported by Honsdorf et al. [18]. Plants were pre-grown on static benches within the Smarthouse and watering was performed manually to allow optimal germination and seedling establishment. Soil mix used for the experiment was 50% cocopeat mixed with 50% clay loam. At 31 days after planting (DAP), the pots were transferred to the automated section of the Smarthouse where each pot was placed onto a cart on a conveyor belt. On the first day of automated phenotyping, all pots were watered to a gravimetric water content of 25%, which is equivelent to 508 g of water per 2030 g of dry soil used for potting. Control pots kept this water content whereas water-limited pots were allowed to dry down to 15% (g/g) water content. The two treatments were maintained until 59 DAP by watering every day.

### Phenotyping

During the period from 32 to 59 DAP when the stress treatment was applied, plant images were captured daily using a LemnaTec 3D Scanalyzer (LemnaTec, GmbH, Wuerselen, Germany). Every day, three RGB pictures were taken of each barley plant, one top view image and two side view images with a 90° horizontal rotation. After separating the plant tissue area from the background, pixel numbers per plant were counted and the pixel sum of the three pictures per plant was used as the total projected shoot area per plant per day (designated as PSA). In 2014, images were taken with 5 megapixel cameras whereas in 2015 and 2016, 8 megapixel cameras were used. 2014 results were scaled accordingly to account for the difference in camera resolution and optics used.

The PSA data for each individual plant was smoothed by fitting a natural cubic spline using the smooth.spline function in R to obtain the shoot area smoothed (SAsm). The SAsm of the last day of imaging (DAP = 59) was used for calculation of best linear unbiased estimates (BLUEs), which was subsequently used for GWAS.

Absolute growth rate (AGR) and relative growth rate (RGR) for each plant were calculated as described in Table 1. Three intervals were identified based on the kinetics of AGRs and RGRs from 32 to 59 DAP where plants were in a particular growth phase, (i.e. constant growth rate, accelerating or decelerating growth). These three intervals were defined as 32–40, 42–50, and 52–59 DAP and the AGRs and RGRs for the three intervals were calculated. As a result, for each trait, a single value for each cart was obtained. AGRs for the whole period of imaging are essentially the same as the end-of imaging value for the trait and so it was redundant to include them. Moreover, two other traits were extracted from the images including caliper length integral (CL) and convex hull area integral (CHA). At the end of the experiment, barley plants were harvested and above ground biomass, tiller number (TN), and plant height (HEI) were determined. Plant growth stage was recorded at the completion of the 2015 and 2016 experiments using the Zadok scale [80]. This is the time when destructive measurements were conducted, approximately 2 months after planting. Fresh biomass (FW) was weighed and, subsequently, oven dried to constant weight to determine dry biomass (DW). Water use efficiency (WUE) was calculated by dividing dry biomass at the end of the experiment by the total amount of water added during the 4 weeks in the Smarthouse [mg/g water]. A summary of trait definitions is given in Table 1.

**Table 1:** List of measured traits

	Trait	Abbreviation	Unit	Method of measurement
Imaging parameters				
1	Shoot area smoothed	SAsm	kPix^a^	Smoothing spline fit to total projected shoot area data for each plant
2	Absolute growth rate smoothed 32–40	AGR32	kPix/d^b^	Difference in smoothed Shoot Area between days 32th and 40th after planting, divided by the length of the period
3	Absolute growth rate smoothed 42–50	AGR42	kPix/d	Difference in smoothed Shoot Area between days 42th and 50th after planting, divided by the length of the period
4	Absolute growth rate smoothed 52–59	AGR52	kPix/d	Difference in smoothed Shoot Area between days 52th and 59th after planting divided by the length of the period
5	Relative growth rate smoothed 32–40	RGR32	d^−1^	Difference in the logarithm of the smoothed Shoot Area at days 32th and 40th after planting, divided by the length of the period
6	Relative growth rate smoothed 42–50	RGR42	d^−1^	Difference in the logarithm of the smoothed Shoot Area at days 42th and 50th after planting, divided by the length of the period
7	Relative growth rate smoothed 52–59	RGR52	d^−1^	Difference in the logarithm of the smoothed Shoot Area at days 52th and 59th after planting, divided by the length of the period
8	Convex hull area integral^c^	CHA	kPix	Smallest geometrical object without concave parts that covers whole plant, top view image
9	Caliper length integral	CL	kPix	Max. distance between two points on the object boundary, top view image
Harvest parameters				
10	Fresh weight	FW	g	Weight of fresh biomass per pot
11	Dry weight	DW	g	Weight of oven dried biomass per pot
12	Plant height	HEI	cm	Plant height measured from bottom to leaf tip
13	Tiller number	TN		Number of tillers per pot
Indices				
14	Water use efficiency	WUE	g/g water	Harvested dry biomass per plant/total amount of irrigation water

^a^*kPix* Kilo pixel

^b^*kPix/d* Kilo pixel/day

^c^integral: calculated for the length of entire experiment

### Statistical analysis of phenotypic data

A two-step analysis was performed in analysing the phenotypic data: (i) a mixed model analysis was performed on the data for each year to produce spatially adjusted BLUEs for each combination of the genotypes and treatments; (ii) a mixed model analysis of the BLUEs from (i) were combined over the 3 years and a mixed model analysis performed to produce BLUEs adjusted for differences between the years.In addition, a genetic analysis was carried out on the data for each year using a mixed model in which the genotypic effects are assumed random for each treatment and heritability coefficients are computed from the analysis using the method described by Cullis et al. [81].

The mixed model used for the first step was similar to that used in [51] and was as follows:

$$ \mathbf{y}=\mathbf{X}\boldsymbol{\upbeta } +\mathbf{Zu}+\mathbf{e}, $$

where **y** is the response vector of values for the trait being analysed; **β** is the vector of fixed effects; **u** is the vector of random effects; and **e** is the vector of residual effects. **X** and **Z** are the design matrices corresponding to **β** and **u** respectively.

The fixed-effect vector **β** partitioned as $\left[\mu\ {\boldsymbol{\upbeta}}_{\mathrm{S}}^{\top }\ {\boldsymbol{\upbeta}}_{\mathrm{S}:\mathrm{cL}}^{\top }\ {\boldsymbol{\upbeta}}_{\mathrm{S}:\mathrm{cM}}^{\top }\ {\boldsymbol{\upbeta}}_{\mathrm{C}}^{\top }\ {\boldsymbol{\upbeta}}_{\mathrm{G}}^{\top }\ {\boldsymbol{\upbeta}}_{\mathrm{T}}^{\top }\ {\boldsymbol{\upbeta}}_{\mathrm{C}:\mathrm{T}}^{\top }\ {\boldsymbol{\upbeta}}_{\mathrm{G}:\mathrm{T}}^{\top }{\boldsymbol{\upbeta}}_{\mathrm{S}:\mathrm{cZ}}^{\top}\right]$, where (i) *μ* is the overall mean (ii) ${\boldsymbol{\upbeta}}_{\mathrm{S}}^{\top }$ are the Smarthouse effects, (iii) ${\boldsymbol{\upbeta}}_{\mathrm{S}:\mathrm{cL}}^{\top }$ and ${\boldsymbol{\upbeta}}_{\mathrm{S}:\mathrm{cM}}^{\top }$ are the linear trend coeficients for the centred, numerical variables cMainPosn (east-west) and cLanes (north-south) in each Smarthouse,(iv) ${\boldsymbol{\upbeta}}_{\mathrm{C}}^{\top }$, ${\boldsymbol{\upbeta}}_{\mathrm{G}}^{\top }$, ${\boldsymbol{\upbeta}}_{\mathrm{T}}^{\top }$, ${\boldsymbol{\upbeta}}_{\mathrm{C}:\mathrm{T}}^{\top }$ and ${\boldsymbol{\upbeta}}_{\mathrm{G}:\mathrm{T}}^{\top }$ are the subvectors for the effects of the Checks (C), the Genotypes (G), the Treatments (T), the Check-by-Treatment interactions (C:T), and the Genotype-by-Treatment interactions (G:T), and (v) ${\boldsymbol{\upbeta}}_{\mathrm{S}:\mathrm{cZ}}^{\top }$ are the linear regression effects in each Smarthouse for the relationship with the numeric Zadok’s growth stage scores at the end of imaging, which is centred at growth stage 33 (not included for Year 1).

The random effects vector **u** is partitioned as $\left[{\mathbf{u}}_{\mathrm{spl}\left(\mathrm{S}:\mathrm{cL}\right)}^{\top }\ {\mathbf{u}}_{\mathrm{spl}\left(\mathrm{S}:\mathrm{cM}\right)}^{\top }\ {\mathbf{u}}_{\mathrm{S}:\mathrm{Z}:\mathrm{M}}^{\top}\right]$ where the **u**s are the subvectors of the coefficients of the spline basis functions for fitting curved trends within each Smarthouse over Lanes (spl(S:cL)), the coefficients of the spline basis functions for fitting curved trends within each Smarthouse over the east-west positions of the main plots (spl(S:cM)) and the random main-plot effects within each Zone in each Smarthouse (S:Z:M). The design matrices **X** and **Z** are partitioned to conform to the partitioning of **β** and **u**, respectively. It is assumed that each subvector of random effects, **u**_*i*_, is distributed *N*(**0**_*m*_, *σ*_*i*_**I**_*m*_), where **0**_*m*_ is the m-vector of zeroes, *σ*_*i*_ is the variance of the ith set of random effects, **I**_*m*_ is the identity matrix of order *m*, and *m* is the order of **u**_*i*_. Further, with the distribution of the residual effects **e** are assumed to be:

$$ \boldsymbol{N}\left({\mathbf{0}}_{\mathbf{1}\mathbf{0}\mathbf{56}},\left[\begin{array}{cccc}{\boldsymbol{\sigma}}_{\mathbf{1}\boldsymbol{w}}^{\mathbf{2}}& \mathbf{0}& \mathbf{0}& \mathbf{0}\\ {}\mathbf{0}& {\boldsymbol{\sigma}}_{\mathbf{1}\mathbf{\ell}}^{\mathbf{2}}& \mathbf{0}& \mathbf{0}\\ {}\mathbf{0}& \mathbf{0}& {\boldsymbol{\sigma}}_{\mathbf{2}\boldsymbol{w}}^{\mathbf{2}}& \mathbf{0}\\ {}\mathbf{0}& \mathbf{0}& \mathbf{0}& {\boldsymbol{\sigma}}_{\mathbf{2}\mathbf{\ell}}^{\mathbf{2}}\end{array}\right]\mathbf{\bigotimes}{\mathbf{I}}_{\mathbf{2}\mathbf{64}}\right) $$

where ${\sigma}_{1w}^2$, ${\sigma}_{2\ell}^2$, ${\sigma}_{1w}^2$ and ${\sigma}_{2\ell}^2$ are, respectively, the variances of the residuals for the pots in control conditions in the two Smarthouses and the pots in the control conditions in the two Smarthouses; it is assumed that the data in **y** are ordered to conform with the order as the variances. This model allows for the residuals for the two treatments to have different residual variances in the two Smarthouses. Residual-versus-fitted-values and normal probability plots were obtained and inspected for all traits in all years and none revealed any deficiencies in the models used. Except for ${\sigma}_{\mathrm{S}:\mathrm{Z}:\mathrm{M}}^2$, each of the variance components and the need for unequal residual variances was tested via REML ratio tests with *α* = 0.05. If the curved trends were not significant then Wald F tests, employing degrees of freedom calculated using the Kenward-Rogers method, were used to determine if there was any trend at all. These mixed model analyses were carried out using the packages asreml [82], with REML used as the method of estimation, and asremlPlus [83] in the R statistical computing environment (R Core Team, 2016). The BLUEs were obtained using the resulting model.

For the second step in the analysis, the 3 years of genotype BLUEs for a treatment were combined and subject to a mixed model analysis using PROC MIXED in SAS, with genotype treated as a fixed effect and year as a random effect. The BLUEs from this analysis were obtained using the LSMEANS statement in PROC MIXED, with variance components estimated using type3; the BLUEs for the HEB lines formed the data for the GWAS analysis..

### Genome wide association study (GWAS)

A set of 5709 barley Illumina 9 K iSelect SNPs previously mapped in the HEB-25 population [35] was available for GWAS. Of these, a set of 5333 SNPs with a minor allele frequency greater than 1% was utilized for GWAS in this study. The differentiation of the SNP genotypes was based on an identity-by-state (IBS) approach described by Maurer et al. [35].

GWAS was performed using a multiple linear regression model referred to as model-A by Liu et al. [84], where: $\mathbf{y}=\mu +{\mathbf{X}}_{{\mathrm{SNP}}_{\mathrm{IBS}}}{\boldsymbol{\upbeta}}_{{\mathrm{SNP}}_{\mathrm{IBS}}}+\mathbf{e}$. This multiple regression model takes into account a quantitative SNP effect in addition to quantitative cofactors that control both population structure and genetic background [85]. Cofactor selection was carried out on this model and included all SNPs simultaneously by applying PROC GLMSELECT in SAS. SNPs were allowed to enter or leave the model based on a SNP’s *p*-value < 0.001 for the marginal F-test. To reduce false positives and increase the robustness of the GWAS results, a five-fold cross-validation was run 20 times and parent-specific marker effects were estimated. The procedure of cross validation and estimation of parent-specific QTL effect was described in detail by Maurer et al. [36]. Markers that were detected 20 times out of 100 cross-validation runs were accepted as putative QTL. Candidate genes for QTL detected by GWAS were identified using the BARLEYMAP pipeline [86]. In addition we compared the genomic position of QTL with the position of known flowering/developmental and plant architecture genes in barley summarized by Alqudah et al. [87] and Alqudah et al. [59] as these two papers used the same genetic map and markers as in this study. Genes were suggested as candidates if they were within 4 cM of a QTL.

## Additional files

Additional file 1: **Figure S1.** Relative growth rate (RGR), absolute growth rate (AGR), and shoot area smoothed (SAsm) of all plants grown within the drought stress experiments across 3 years (2014–2016) at the Plant Accelerator, University of Adelaide. The solid line represents the average of control conditions (cyan) and drought conditions (red). (PDF 696 kb)

Additional file 2: **Figure S2.** Comparison of temperature recorded inside the north-east (NE) and north-west (NW) Smarthouses during the experimental period in the 3 years from 2014 to 2016 at the Plant Accelerator. A and B. Lineplots showing highest and lowest temperature recorded by sensors for north-east and north-west Smarthouses. C. Lineplot of growing degree days during the course of the experiment for two Smarthouses. (PDF 101 kb)

Additional file 3: **Table S3.** *P* values of all terms in the models for 14 traits across 3 years. (XLSX 15 kb)

Additional file 4: **Table S4.** Summary of simple statistics for the drought stress experiment from 2014 to 2016. (XLSX 21 kb)

Additional file 5: **Figure S6.** Scatter plots for shoot area smoothed (SAsm), dry weight (DW), tiller number (TN), and plant height (HEI) in 3 years from 2014 to 2016. A. Scatter plots and correlation coefficients between the ratio of the phenotypic values in drought stress treatment versus the control control treatment (Ratio_D2W_) and the corresponding phenotypic values in control treatment for shoot area smoothened in 3 years 2014–2016, respectively. B. Scatter plots and correlation coefficients between ratio of the phenotypic values in drought stress treatment versus the control control treatment (Ratio_D2W_) and the corresponding phenotypic values in control treatment for plant height in 3 years 2014–2016, respectively. C. Scatter plots and correlation coefficients between ratio of the phenotypic values in drought stress treatment versus the control control treatment (Ratio_D2W_) and the corresponding phenotypic values in control treatment for dry weight in 3 years 2014–2016, respectively. D. Scatter plots and correlation coefficients between ratio of the phenotypic values in drought stress treatment versus the control control treatment (Ratio_D2W_) and the corresponding phenotypic values in control treatment for tiller number in 3 years 2014–2016, respectively. (PDF 265 kb)

Additional file 6: **Table S6.** Overview of all QTL and their significant effects on 14 traits studied in 25 families of the HEB-25 population. (XLSX 280 kb)

Additional file 7: **Figure S5.** Correlation matrices for 14 traits in each treatment from 2014 to 2016. In the following plots, the distribution of each variable is shown on the diagonal. The bivariate scatter plots with a fitted line are displayed on the bottom of the diagonal. The value of the correlation plus the significance level as stars are displayed on the top of the diagonal. Each significance level is associated to a symbol: *p*-values (0, 0.001, 0.05, 0.01) < => symbols (“***”, “**”, “*”). (PDF 516 kb)

Additional file 8: Design for the drought stress experiment on the NAM Barley lines. (PDF 630 kb)

## Abbreviations

AGR: Absolute growth rate
BLUEs: Best linear unbiased estimators
CHA: Convex hull area
CL: Caliper length
DAP: Days after planting
DW: Dry weight
FW: Fresh weight
GWAS: Genome-wide association study
HEB: Halle exotic barley
HEI: Plant height
NAM: Nested association mapping
NE: North East
NW: North West
QTL: Quantitative trait loci
RGR: Relative growth rate
SAsm: Shoot area smoothed
SNPs: *Single nucleotide polymorphisms*
TN: Tiller number
Vp: phenotypic variance
WUE: Water use efficiency
