# Supplementary figures and images for: Genome-wide association of barley plant growth under drought stress using a nested association mapping population
Source: BMC Plant Biol. 2019 Apr 11;19:134. doi: 10.1186/s12870-019-1723-0 (PMC6458831; doi:10.1186/s12870-019-1723-0)

Relative  
growth  
rate

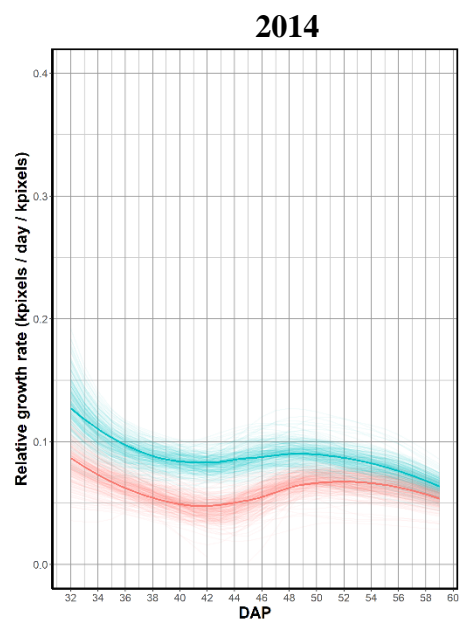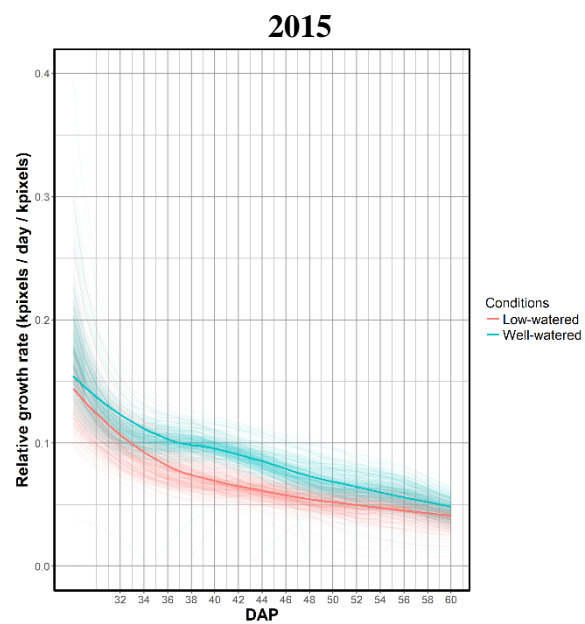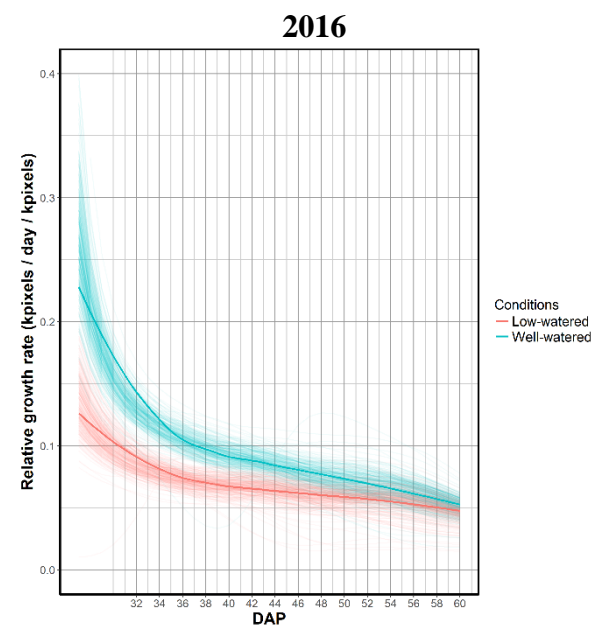

Absolute  
growth  
rate

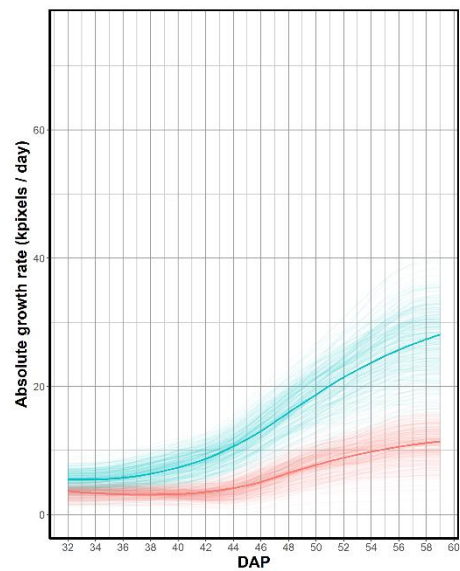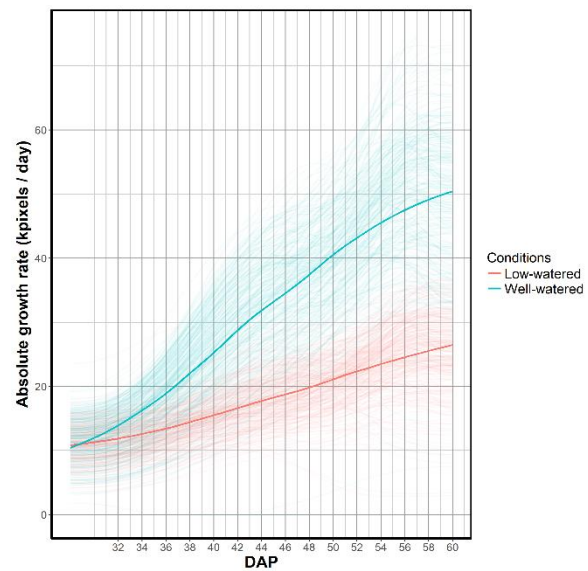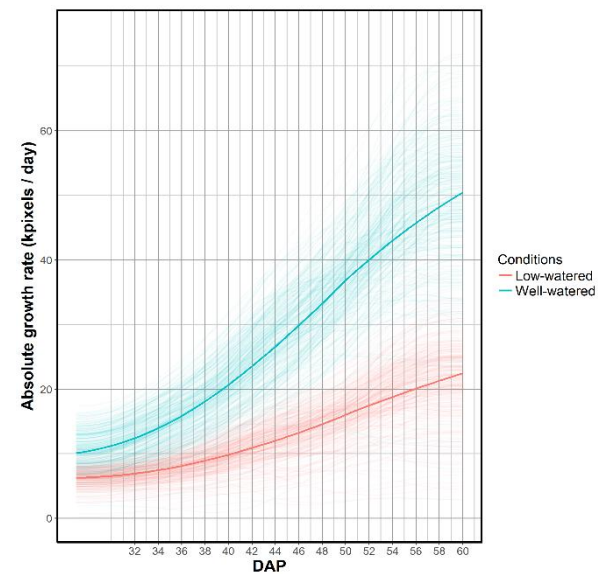

Shoot  
Area  
smoothed

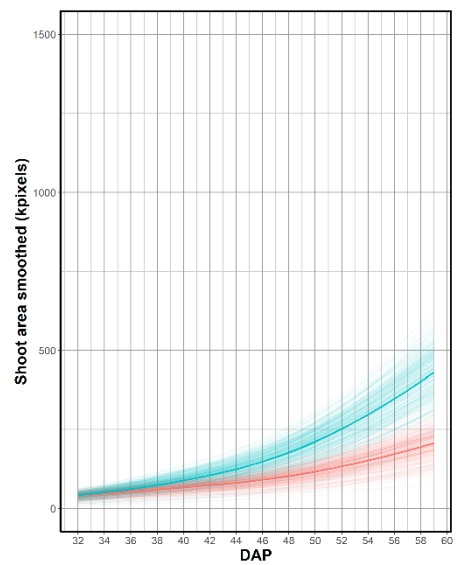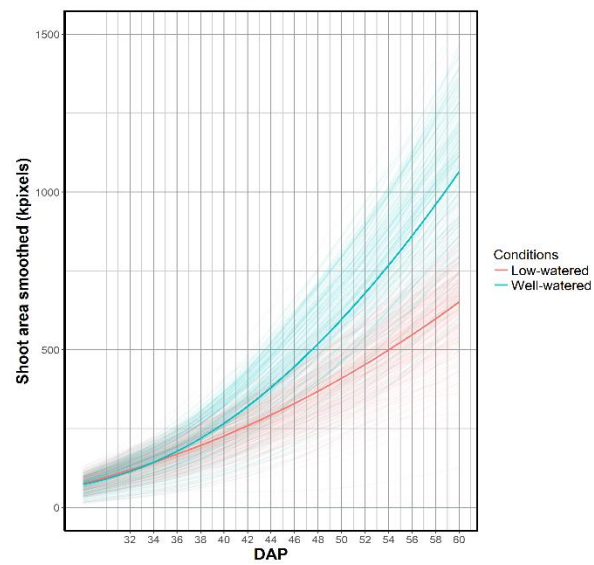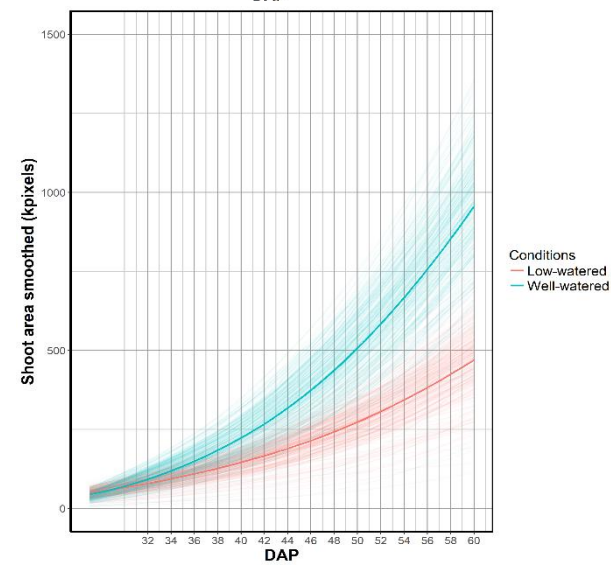

Supplement: Supplementary file 1 — Figure S1. Relative growth rate (RGR), absolute growth rate (AGR), and shoot area smoothed (SAsm) of all plants grown within the drought stress experiments across 3 years (2014–2016) at the Plant Accelerator, University of Adelaide. The solid line represents the average of control conditions (cyan) and drought conditions (red). (PDF 696 kb) [file 12870_2019_1723_MOESM1_ESM.pdf]

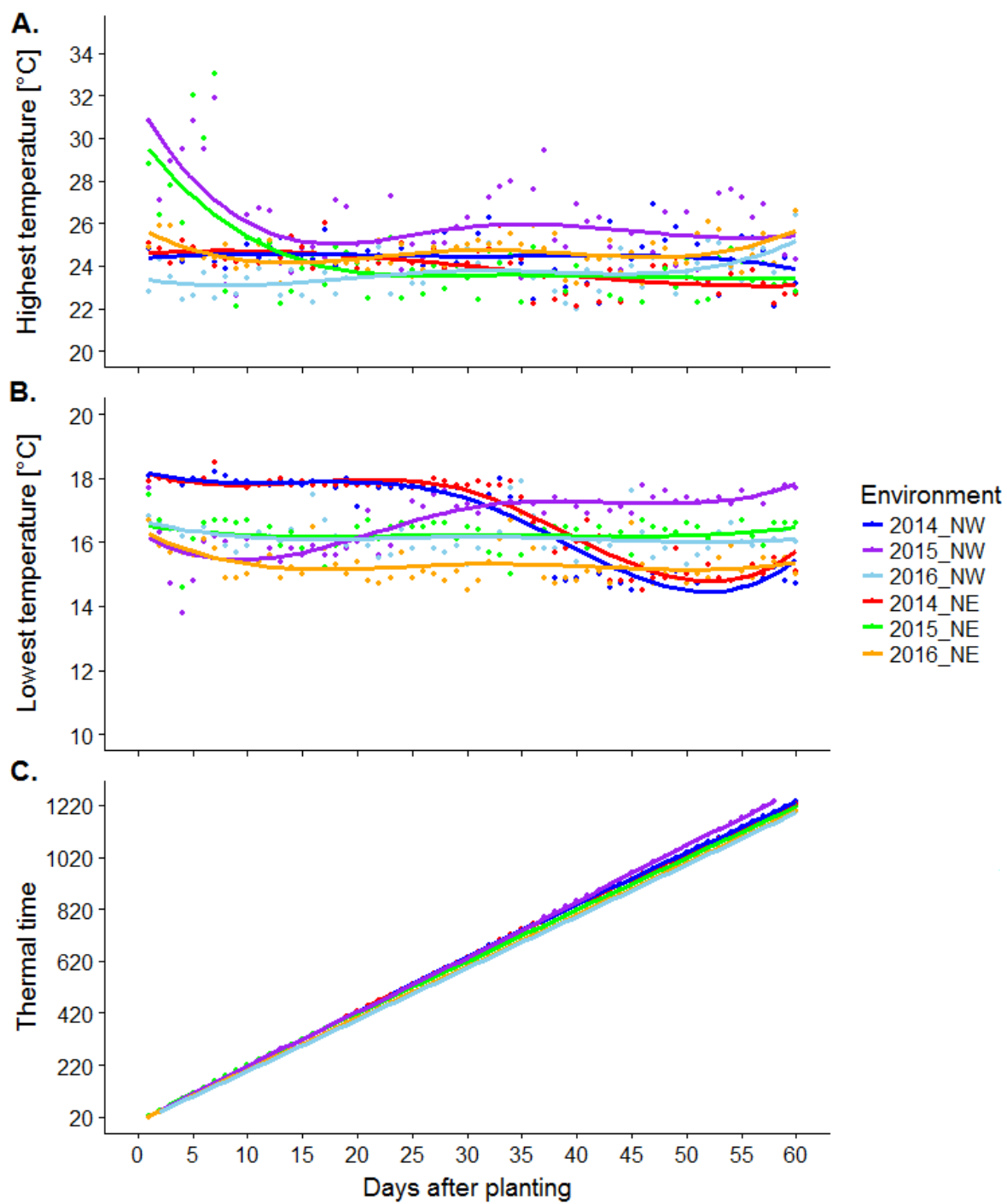

Supplement: Supplementary file 2 — Figure S2. Comparison of temperature recorded inside the north-east (NE) and north-west (NW) Smarthouses during the experimental period in the 3 years from 2014 to 2016 at the Plant Accelerator. A and B. Lineplots showing highest and lowest temperature recorded by sensors for north-east and north-west Smarthouses. C. Lineplot of growing degree days during the course of the experiment for two Smarthouses. (PDF 101 kb) [file 12870_2019_1723_MOESM2_ESM.pdf]

A

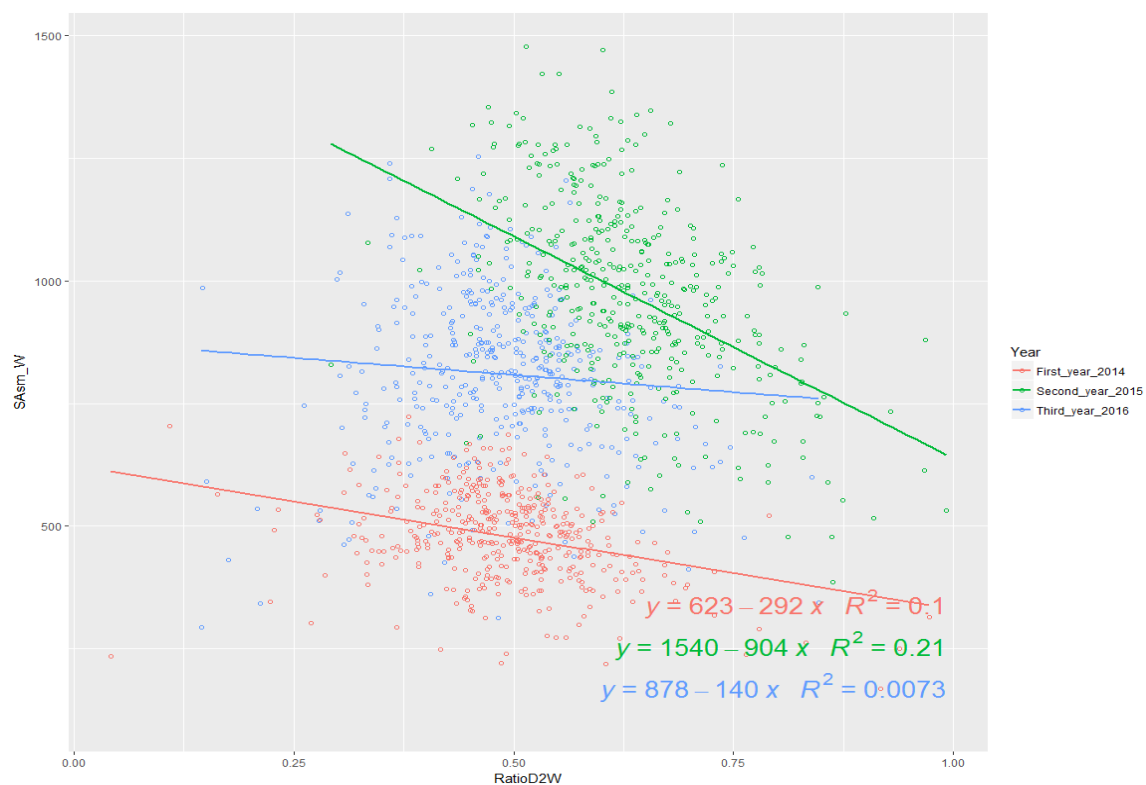

B

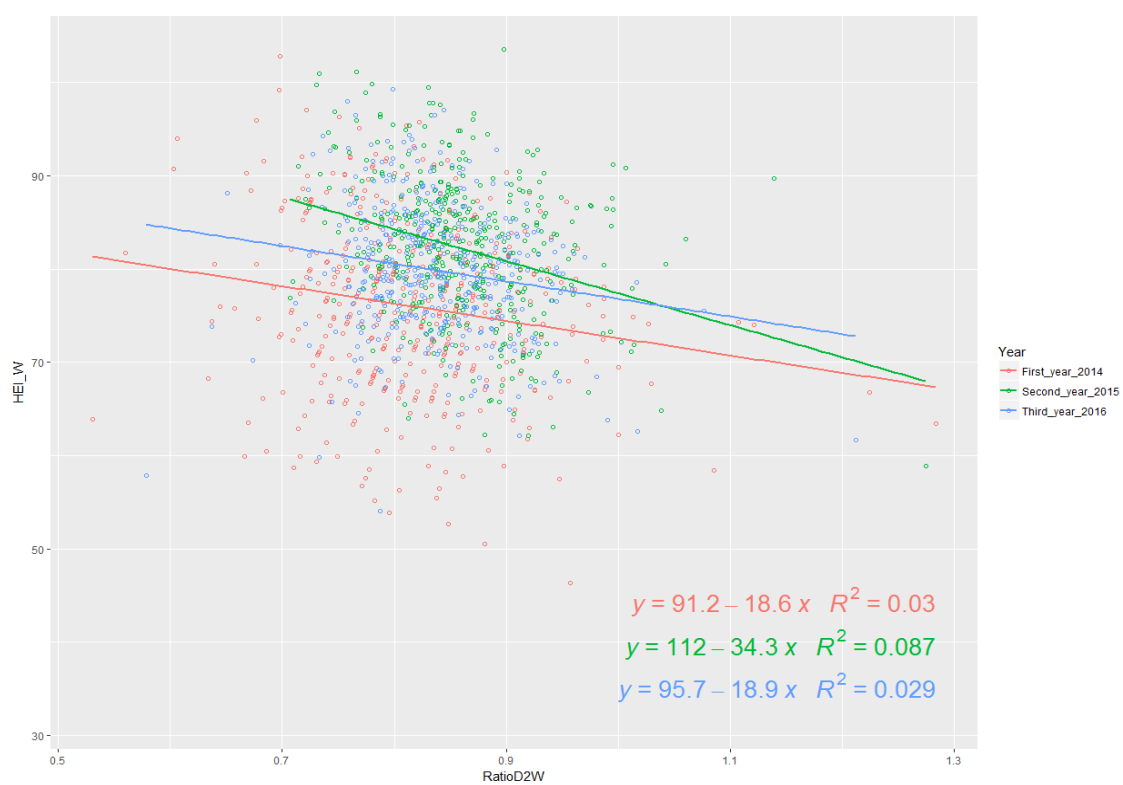

C

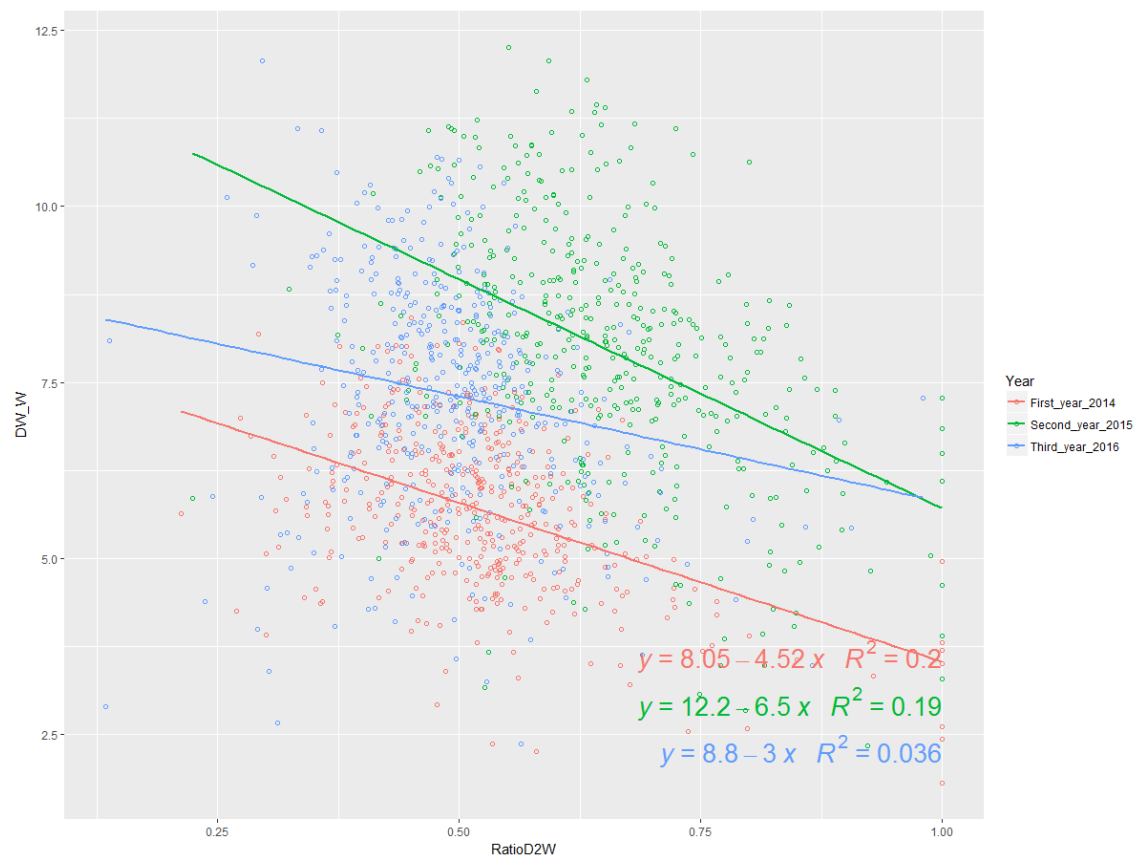

D

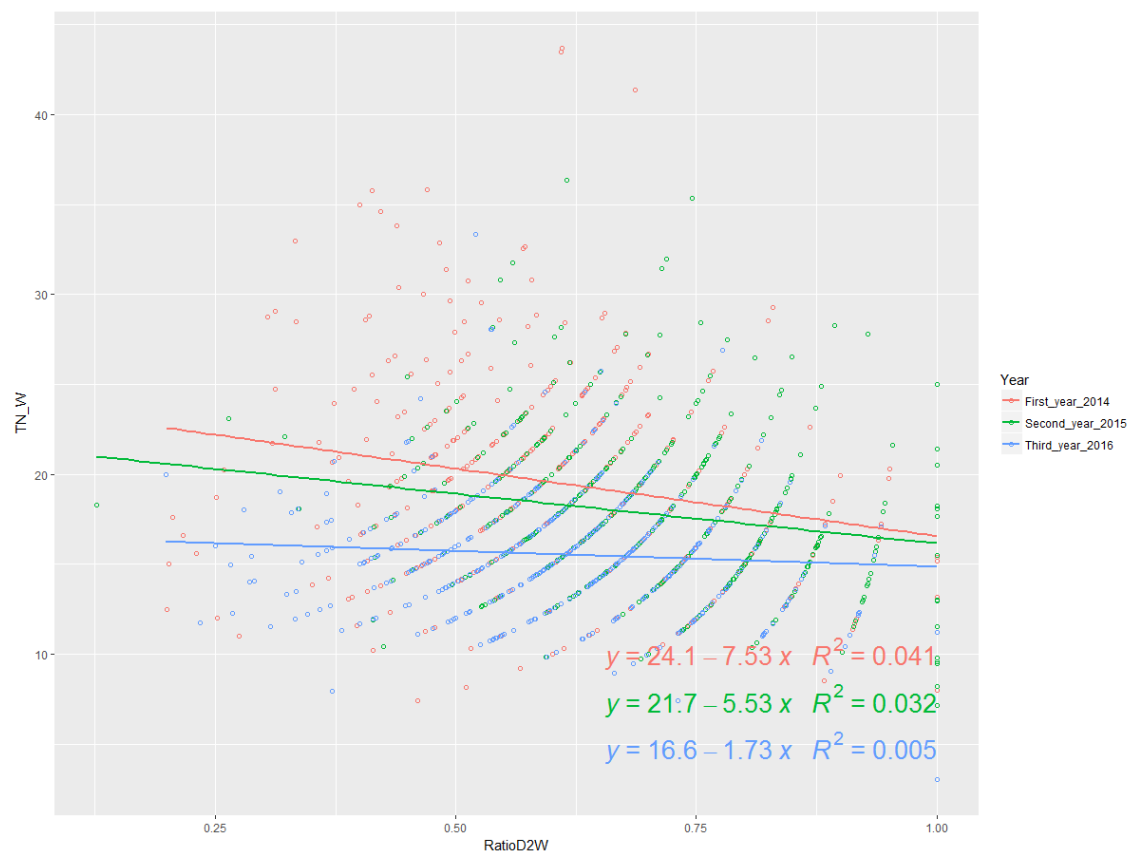

Supplement: Supplementary file 5 — Figure S6. Scatter plots for shoot area smoothed (SAsm), dry weight (DW), tiller number (TN), and plant height (HEI) in 3 years from 2014 to 2016. A. Scatter plots and correlation coefficients between the ratio of the phenotypic values in drought stress treatment versus the control control treatment (RatioD2W) and the corresponding phenotypic values in control treatment for shoot area smoothened in 3 years 2014–2016, respectively. B. Scatter plots and correlation coefficients between ratio of the phenotypic values in drought stress treatment versus the control control treatment (RatioD2W) and the corresponding phenotypic values in control treatment for plant height in 3 years 2014–2016, respectively. C. Scatter plots and correlation coefficients between ratio of the phenotypic values in drought stress treatment versus the control control treatment (RatioD2W) and the corresponding phenotypic values in control treatment for dry weight in 3 years 2014–2016, respectively. D. Scatter plots and correlation coefficients between ratio of the phenotypic values in drought stress treatment versus the control control treatment (RatioD2W) and the corresponding phenotypic values in control treatment for tiller number in 3 years 2014–2016, respectively. (PDF 265 kb) [file 12870_2019_1723_MOESM5_ESM.pdf]
