# Supplementary material for: Genome-wide association of barley plant growth under drought stress using a nested association mapping population
Source: BMC Plant Biol. 2019 Apr 11;19:134. doi: 10.1186/s12870-019-1723-0 (PMC6458831; doi:10.1186/s12870-019-1723-0)

a. Year 2014, drought stress treatment

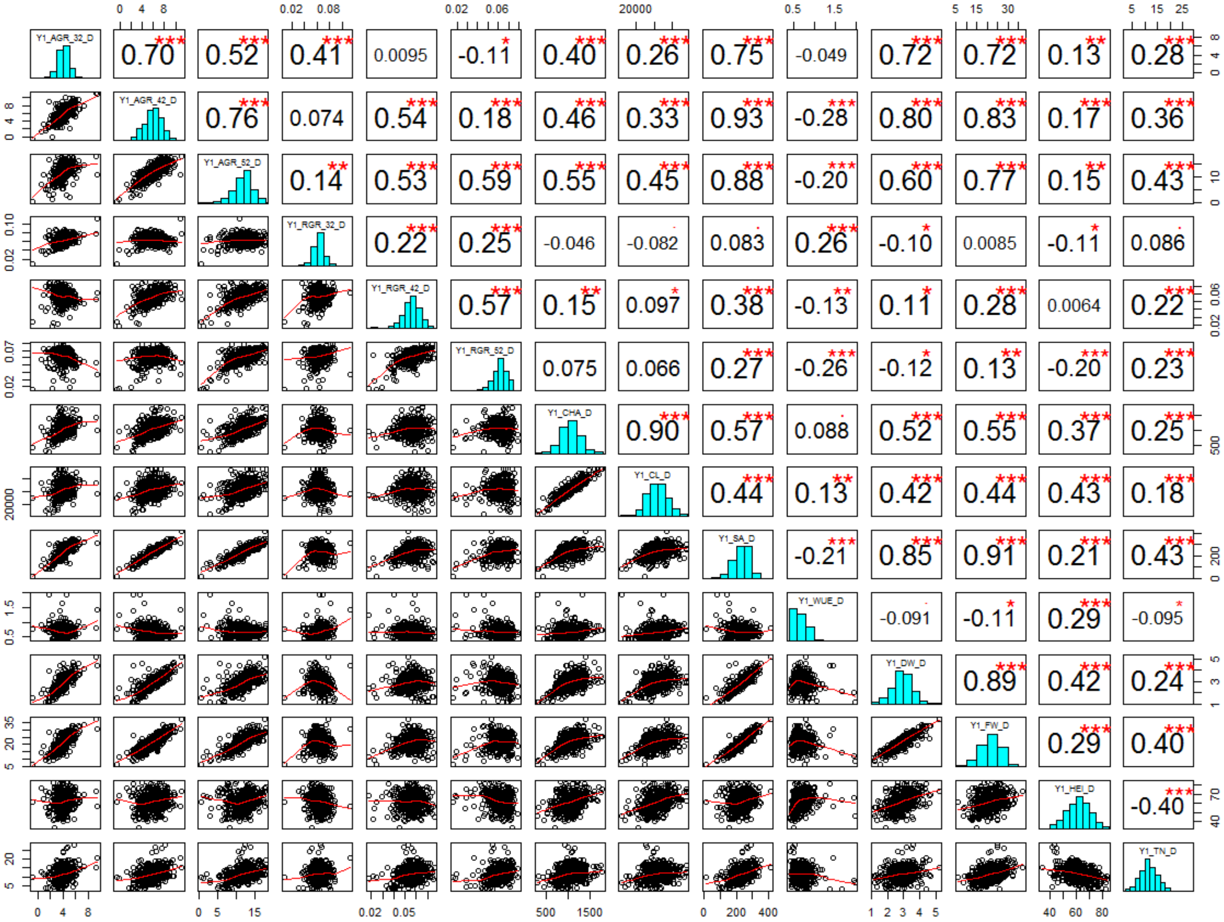

b. Year 2014, control treatment

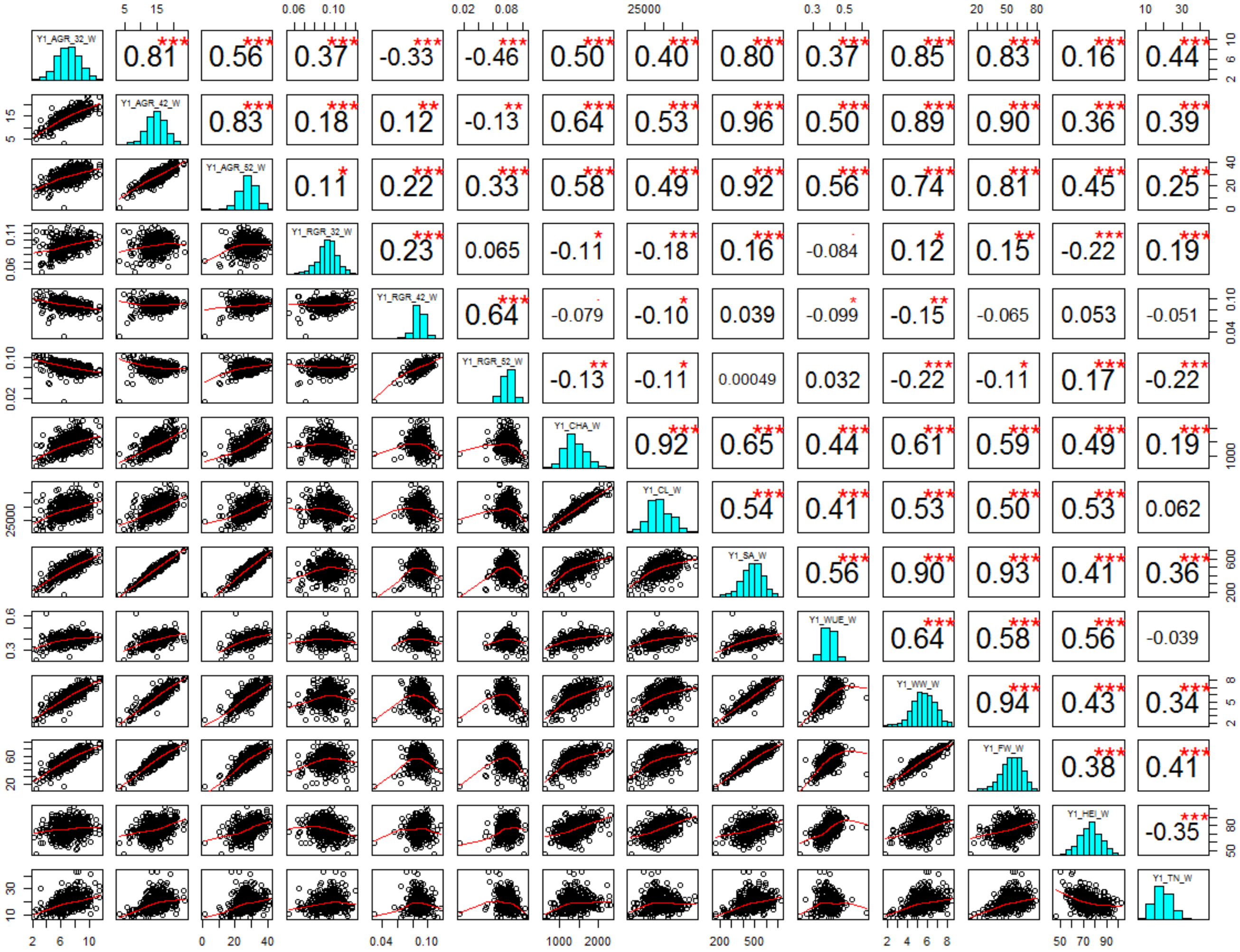

c. Year 2015, drought stress treatment

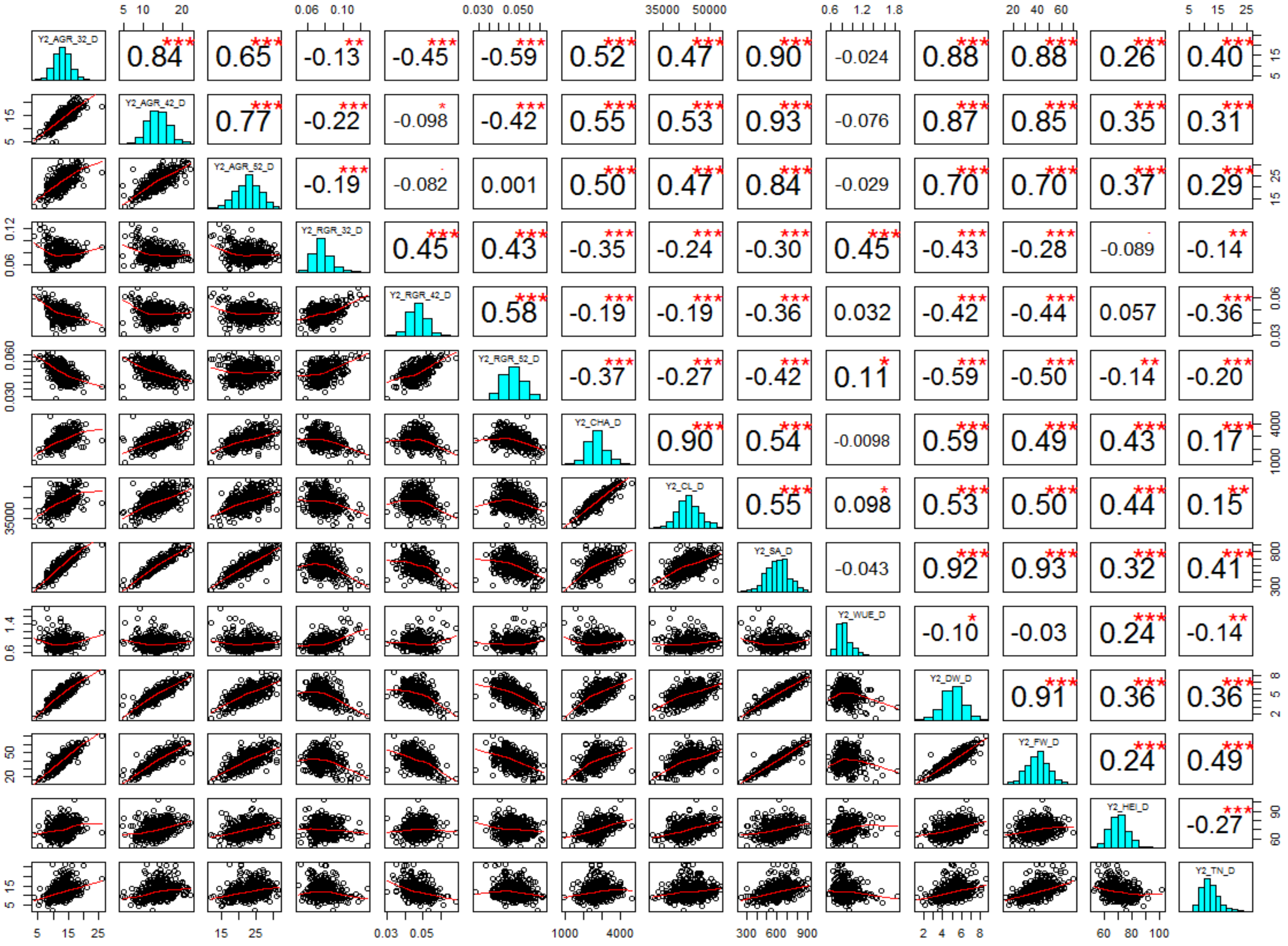

d. Year 2015, control treatment

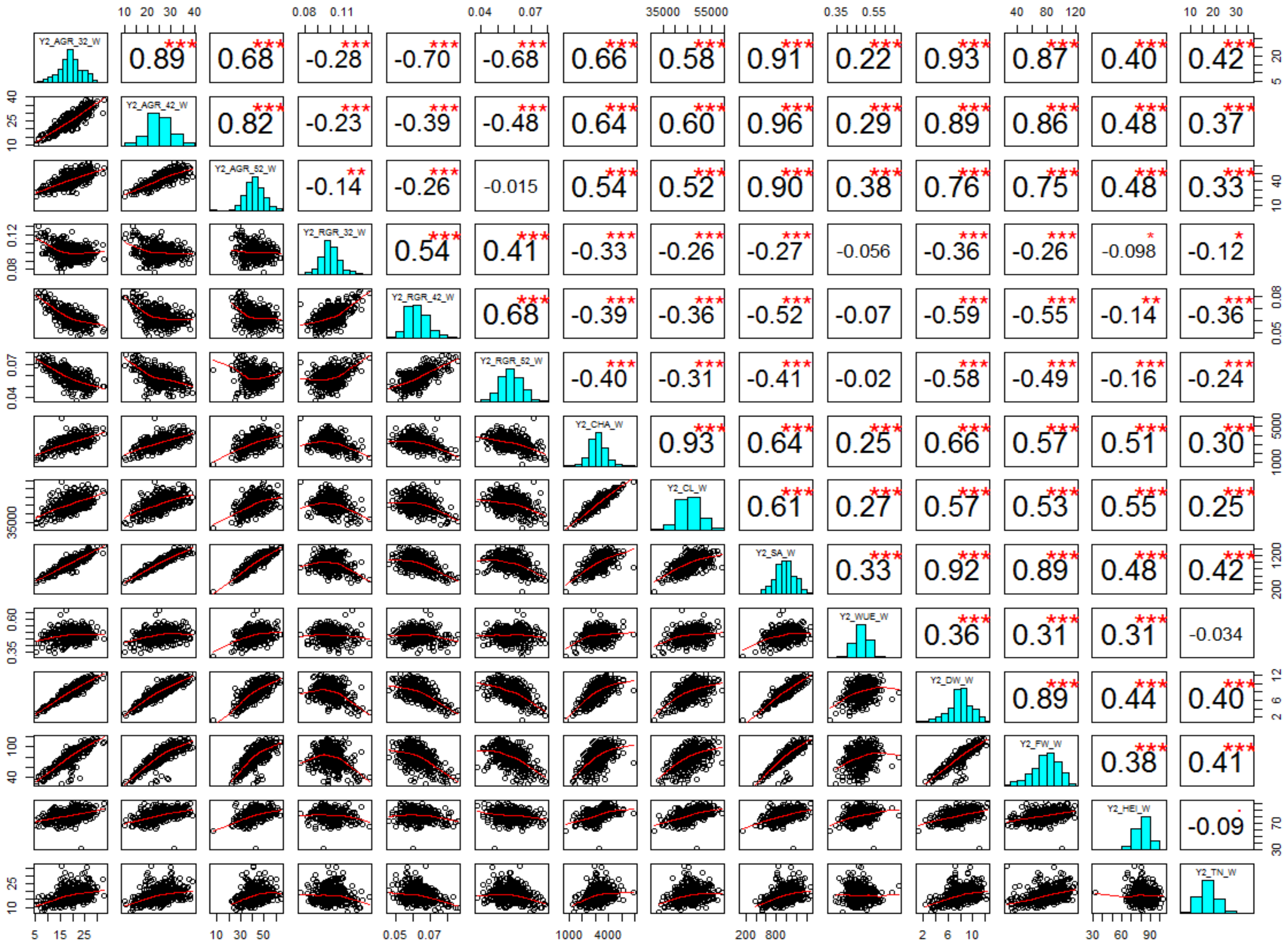

e. Year 2016, drought stress treatment

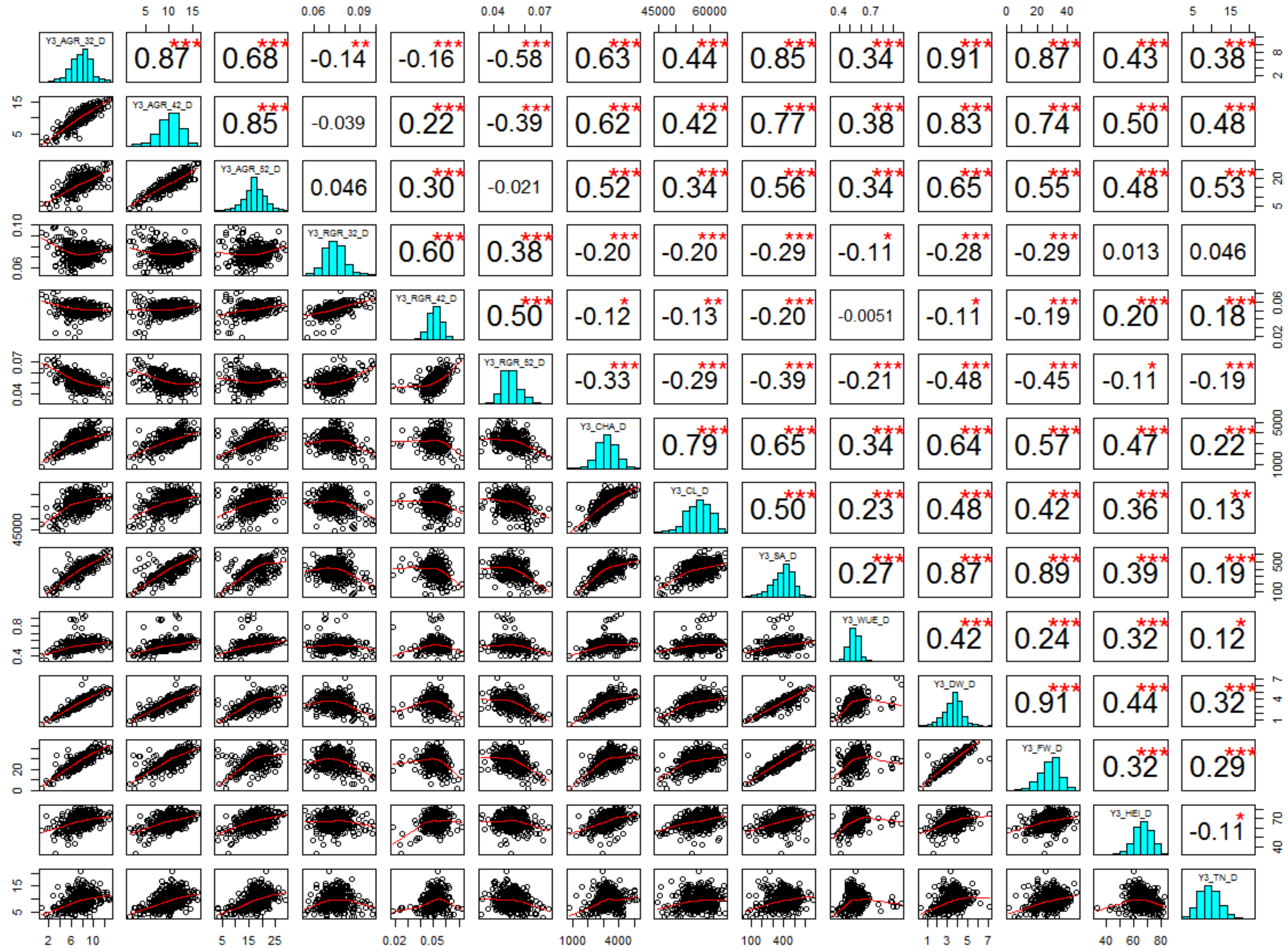

f. Year 2016, control treatment

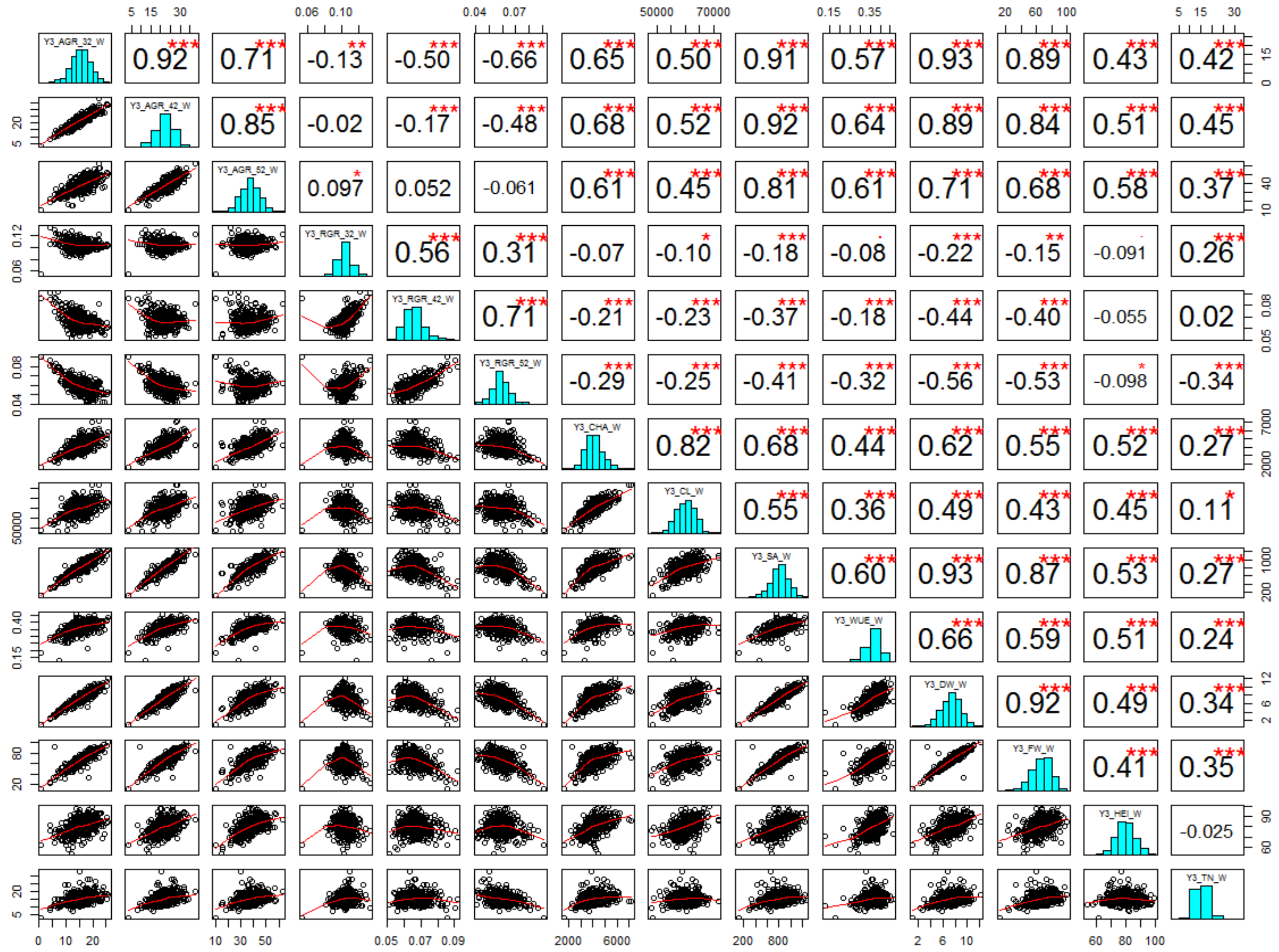

Supplement: Supplementary file 7 — Figure S5. Correlation matrices for 14 traits in each treatment from 2014 to 2016. In the following plots, the distribution of each variable is shown on the diagonal. The bivariate scatter plots with a fitted line are displayed on the bottom of the diagonal. The value of the correlation plus the significance level as stars are displayed on the top of the diagonal. Each significance level is associated to a symbol: p-values (0, 0.001, 0.05, 0.01) < => symbols (“***”, “**”, “*”). (PDF 516 kb) [file 12870_2019_1723_MOESM7_ESM.pdf]
