# Supplementary material for: Genome-wide association of barley plant growth under drought stress using a nested association mapping population
Source: BMC Plant Biol. 2019 Apr 11;19:134. doi: 10.1186/s12870-019-1723-0 (PMC6458831; doi:10.1186/s12870-019-1723-0)
Supplement: Supplementary file 8 — Design for the drought stress experiment on the NAM Barley lines. (PDF 630 kb) [file 12870_2019_1723_MOESM8_ESM.pdf]

Experimental design for the drought experiment on the NAM Barley lines

Chris Brien
June 19, 2014

This experiment investigates 1343 of 1419 offspring of crosses between the Barley line Barke and 25 donor Barley lines, the resulting collection being referred to as the NAM collection. The experiment includes the 1343 offspring, Barke and Navigator. All lines, except Barke and Navigator, are unreplicated; Navigator is replicated 72 times and Barke 8 or 10 times in each Smarthouse run. The experiment involves 3 consecutive runs in which the NW and NE Smarthouses are used. In each Smarthouse, 24 Lanes by 22 (2-23) Positions are utilized.

At each Position there is a cart that contains a pot with a single plant. Pairs of positions are to have two conditions, well-watered and low-watered, randomized to the two positions. Lines are allocated to the $24 \times 11 = 264$ pairs, making a total of $6 \times 264 = 1584$ pairs available for allocating lines. Of the 264 pairs available in a Smarthouse, 36 are to have a check line allocated to them, 224 (or 223 for the first run in the NW Smarthouse) are to have 224 (or 223) crosses allocated to them and the remainder (4 or 5) have the recipient line, Barke, assigned to them.

The 1343 different crosses are those that were used in the a similar experiment investigating the effect of salt on them. In the current experiment, the donor lines that were included in the salt experiment were omitted and replaced with Barke. In the salt experiment, the 224 (or 223) unreplicated lines in a Smarthouse run are lines from 4 (or 5 for the first run in the NW Smarthouse) donors. Up to 5 lines were randomly omitted from each donor family so that all offspring from a family occurred in the same Smarthouse run.

Each Smarthouse is divided into 6 Zones each comprising 4 Lanes by 22 Positions, because sets of 4 lanes were found to be homogeneous in terms of plant growth variability (Brien et al. 2013). The other major source of spatial variation described by Brien et al. (2013) is a gradient from left to right due in part to differences in exposure to air conditions; the direction of the gradient is likely to differ between the Smarthouses because the air conditioners are on opposite sides of each Smarthouse.

The design employed for each Smarthouse run is a split-plot design in which two consecutive carts form a main plot. The main-plot design is an unreplicated design with replicated check and recipient lines, Navigator and Barke. In order to deal with the anticipated spatial variation, lines are allocated to main plots using a blocked, row-and-column design, the blocks being the zones. A feature of the main-plot design is that, for the check lines, (i) there are 6 main plots in each Zone, and (ii) there are 3 or 4 main plots in each column; the Barke main plot are similarly distributed across Zones and columns. The subplot design merely randomizes Conditions (well-, low-watered) to the two carts in each main plot. The main plot design was generated using DiGGer (Coombes, 2009) and the

subplot randomization was done using dae (Brien, 2011), packages for the R statistical computing environment (R Development Core Team, 2014).

The resulting conveyor layouts are given below. An Excel file corresponding to this design is separately provided.

Also supplied at the end of the document is a proposed table layout for the germinations and initial growth periods.

The model to analyse responses that provide a single value for each cart in this experiment is:

$$\begin{aligned} \text{E[Y]} &= \text{Zone} + \text{spl(xMainPosition)} + \text{Lines} * \text{Condition} \\ \text{var[Y]} &= \text{Zones:Mainplots} + \text{idh(Condition):Zones:Mainplots} \end{aligned}$$

where xMainPosition is a centred, numeric variable indexing the Positions of the main plots and allows for a linear trend across the Positions, `spl` indicates a spline is to be fitted and `idh` indicates different variances for the Conditions are to be fitted.

It is noted that, given that there are 36 main plots per Smarthouse-run with the check line and 4 with the recipient line, the main plot residual degrees of freedom in each Smart house-run, remaining after zones and a linear trend have been allowed for, is 32 degrees of freedom. This is considered to be the minimum for these degrees of freedom.

References

Brien, C. J., B. Berger, H. Rabie, and M. Tester (2013) Accounting for variation in designing greenhouse experiments with special reference to greenhouses containing plants on conveyor systems. *Plant Methods* **9**(5): 5.

Brien, C. J. (2011) `dae`: *Functions useful in the design and ANOVA of experiments.* Version 2.1-7. http://cran.r-project.org/

Coombes, N.E. (2009) `Digger` *design search tool in R.* http://www.austatgen.org/files/software/downloads/.

R Development Core Team (2014) *R: A language and environment for statistical computing.* Vienna, Austria: R Foundation for Statistical Computing. http://www.r-project.org.

Run 1 NW Smarthouse conveyor layout of Lines

Run 1 NW Positions

Lines	23	22	21	20	19	18	17	16	15	14	13	12	11	10	9	8	7	6	5	4	3	2
1	487	487	955	955	473	473	1	1	452	452	445	445	1	1	1130	1130	1132	1132	1098	1098	1112	1112
2	1075	1075	1110	1110	457	457	1123	1123	1	1	448	448	479	479	1138	1138	449	449	492	492	1161	1161
3	1	1	1055	1055	967	967	1136	1136	1105	1105	1	1	1163	1163	1158	1158	1074	1074	1079	1079	1162	1162
4	1052	1052	1056	1056	1068	1068	1172	1172	1	1	1033	1033	1114	1114	490	490	1133	1133	2	2	1090	1090
5	458	458	481	481	1077	1077	1092	1092	1118	1118	440	440	1096	1096	1159	1159	1064	1064	1	1	1	1
6	1035	1035	951	951	1101	1101	491	491	1116	1116	1127	1127	1057	1057	463	463	441	441	1087	1087	1	1
7	1152	1152	1039	1039	1097	1097	1038	1038	1109	1109	1	1	1156	1156	1	1	1151	1151	959	959	1086	1086
8	1	1	1028	1028	2	2	1103	1103	1084	1084	1104	1104	1053	1053	1170	1170	1031	1031	447	447	960	960
9	1045	1045	1	1	1134	1134	1115	1115	484	484	467	467	1037	1037	1093	1093	1	1	1061	1061	1154	1154
10	471	471	1059	1059	1	1	1085	1085	462	462	966	966	1147	1147	970	970	1088	1088	1174	1174	1051	1051
11	488	488	1129	1129	1165	1165	969	969	1168	1168	1	1	1124	1124	1058	1058	453	453	1099	1099	1	1
12	483	483	1	1	1050	1050	1107	1107	2	2	469	469	470	470	482	482	472	472	1029	1029	1125	1125
13	1	1	1137	1137	1155	1155	1122	1122	477	477	1046	1046	1095	1095	961	961	1067	1067	968	968	963	963
14	950	950	1080	1080	964	964	1	1	1062	1062	1060	1060	1169	1169	1166	1166	1	1	1032	1032	446	446
15	2	2	1071	1071	954	954	450	450	1027	1027	1135	1135	486	486	1148	1148	454	454	1	1	1126	1126
16	1078	1078	474	474	1121	1121	1034	1034	1	1	460	460	1140	1140	1	1	475	475	953	953	451	451
17	965	965	1063	1063	1073	1073	1091	1091	1144	1144	952	952	1	1	443	443	464	464	459	459	1	1
18	1131	1131	1076	1076	1	1	1054	1054	1117	1117	949	949	1	1	485	485	468	468	1146	1146	1167	1167
19	476	476	2	2	1	1	1030	1030	455	455	1066	1066	1149	1149	1065	1065	962	962	1081	1081	1036	1036
20	1041	1041	1145	1145	1164	1164	1160	1160	480	480	1070	1070	1113	1113	1150	1150	1120	1120	1	1	442	442
21	461	461	1048	1048	1094	1094	478	478	1089	1089	1	1	465	465	1106	1106	1	1	1082	1082	1044	1044
22	1173	1173	1	1	958	958	1141	1141	1102	1102	489	489	1108	1108	957	957	444	444	1171	1171	956	956
23	1157	1157	1100	1100	1043	1043	1	1	1042	1042	1111	1111	1047	1047	456	456	1083	1083	1069	1069	1139	1139
24	1143	1143	1049	1049	1128	1128	1	1	1153	1153	1119	1119	466	466	1	1	1142	1142	1040	1040	1072	1072

The correspondence between the numbers in this figure and the Lines is given in the Lines key sheet of the Excel spreadsheet. The replicated check line Navigator is coloured green, the recipient line Barke is grey and the unreplicated, offspring lines are blue.

Run 1 NW Smarthouse conveyor layout of Conditions

Run 1 NW Positions

Lanes	23	22	21	20	19	18	17	16	15	14	13	12	11	10	9	8	7	6	5	4	3	2
1	2	1	1	2	2	1	2	1	1	2	1	2	1	2	2	1	1	2	2	1	1	2
2	2	1	2	1	1	2	2	1	1	2	1	2	1	2	2	1	1	2	1	2	1	2
3	2	1	1	2	1	2	2	1	1	2	1	2	1	2	2	1	2	1	2	1	2	1
4	1	2	1	2	2	1	2	1	1	2	1	2	2	1	2	1	2	1	1	2	1	2
5	2	1	2	1	2	1	1	2	2	1	1	2	1	2	1	2	2	1	2	1	2	1
6	2	1	1	2	2	1	2	1	2	1	1	2	2	1	1	2	1	2	2	1	1	2
7	1	2	1	2	2	1	1	2	1	2	1	2	2	1	2	1	2	1	1	2	1	2
8	1	2	1	2	1	2	1	2	2	1	1	2	2	1	1	2	1	2	1	2	1	2
9	1	2	2	1	1	2	1	2	2	1	2	1	1	2	2	1	2	1	1	2	1	2
10	1	2	2	1	2	1	2	1	1	2	2	1	2	1	1	2	2	1	2	1	2	1
11	1	2	2	1	1	2	1	2	2	1	1	2	1	2	2	1	1	2	2	1	1	2
12	1	2	2	1	1	2	1	2	1	2	2	1	1	2	1	2	1	2	1	2	1	2
13	1	2	2	1	2	1	1	2	2	1	2	1	2	1	2	1	2	1	1	2	2	1
14	1	2	1	2	2	1	1	2	1	2	1	2	1	2	1	2	2	1	1	2	1	2
15	2	1	2	1	2	1	2	1	1	2	1	2	2	1	1	2	2	1	2	1	2	1
16	1	2	2	1	1	2	2	1	1	2	1	2	2	1	2	1	1	2	2	1	1	2
17	1	2	1	2	2	1	2	1	1	2	2	1	2	1	1	2	1	2	1	2	2	1
18	2	1	2	1	1	2	1	2	1	2	2	1	1	2	1	2	1	2	1	2	1	2
19	2	1	1	2	2	1	1	2	1	2	1	2	2	1	1	2	1	2	2	1	2	1
20	2	1	1	2	1	2	2	1	1	2	2	1	1	2	2	1	1	2	1	2	1	2
21	1	2	2	1	1	2	2	1	2	1	1	2	2	1	1	2	1	2	1	2	1	2
22	1	2	1	2	1	2	2	1	2	1	2	1	1	2	1	2	2	1	1	2	2	1
23	1	2	1	2	2	1	1	2	1	2	1	2	2	1	2	1	2	1	2	1	1	2
24	1	2	2	1	1	2	2	1	2	1	1	2	2	1	1	2	1	2	1	2	1	2

In this diagram, 1 = Well-watered and 2 = Low-watered.

Run 1 NE Smarthouse conveyor layout of Lines

Run 1 NE Positions

Lines	2	3	4	5	6	7	8	9	10	11	12	13	14	15	16	17	18	19	20	21	22	23
1	151	151	177	177	112	112	617	617	665	665	1	1	661	661	634	634	633	633	1	1	157	157
2	619	619	648	648	1	1	610	610	696	696	604	604	197	197	615	615	205	205	712	712	622	622
3	185	185	184	184	2	2	108	108	699	699	138	138	1	1	674	674	714	714	171	171	719	719
4	204	204	718	718	1	1	142	142	641	641	657	657	664	664	136	136	620	620	1	1	107	107
5	124	124	694	694	1	1	109	109	693	693	679	679	677	677	114	114	2	2	173	173	160	160
6	689	689	662	662	211	211	141	141	653	653	672	672	115	115	647	647	1	1	201	201	137	137
7	212	212	706	706	607	607	147	147	1	1	717	717	684	684	150	150	630	630	198	198	1	1
8	625	625	1	1	149	149	143	143	158	158	116	116	681	681	695	695	192	192	676	676	1	1
9	708	708	663	663	119	119	670	670	209	209	1	1	671	671	642	642	611	611	202	202	172	172
10	710	710	628	628	176	176	186	186	206	206	707	707	1	1	656	656	713	713	187	187	613	613
11	632	632	697	697	698	698	637	637	120	120	122	122	145	145	1	1	636	636	180	180	1	1
12	720	720	1	1	668	668	612	612	701	701	1	1	166	166	195	195	167	167	178	178	640	640
13	183	183	208	208	666	666	1	1	1	1	608	608	644	644	716	716	606	606	129	129	182	182
14	188	188	686	686	688	688	1	1	117	117	626	626	669	669	654	654	196	196	643	643	200	200
15	651	651	127	127	614	614	1	1	627	627	148	148	2	2	175	175	113	113	130	130	111	111
16	1	1	125	125	616	616	190	190	609	609	110	110	659	659	683	683	128	128	1	1	687	687
17	210	210	1	1	179	179	629	629	194	194	667	667	1	1	165	165	621	621	139	139	721	721
18	702	702	126	126	193	193	623	623	146	146	690	690	638	638	1	1	673	673	191	191	704	704
19	1	1	164	164	649	649	631	631	618	618	174	174	203	203	1	1	140	140	168	168	660	660
20	646	646	703	703	680	680	705	705	1	1	132	132	121	121	153	153	682	682	692	692	189	189
21	181	181	199	199	155	155	2	2	645	645	163	163	605	605	624	624	1	1	675	675	169	169
22	1	1	133	133	711	711	715	715	1	1	159	159	152	152	650	650	134	134	162	162	156	156
23	131	131	700	700	658	658	685	685	635	635	1	1	652	652	144	144	655	655	161	161	639	639
24	170	170	709	709	135	135	123	123	207	207	118	118	678	678	1	1	1	1	691	691	154	154

The correspondence between the numbers in this figure and the Lines is given in the Lines key sheet of the Excel spreadsheet. The replicated check line Navigator is coloured green, the recipient line Barke is grey and the unreplicated, offspring lines are blue.

Run 1 NE Smarthouse conveyor layout of Conditions

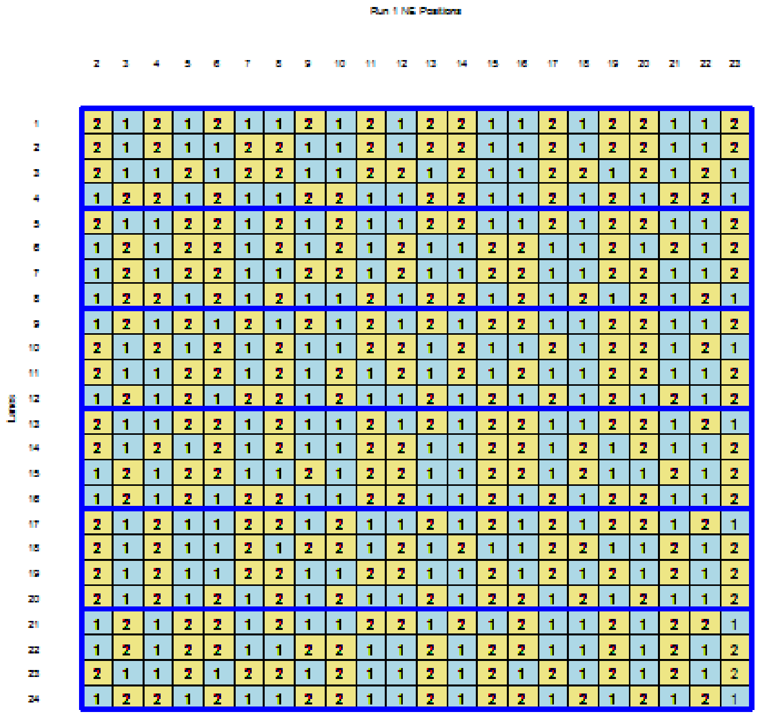

In this diagram, 1 = Well-watered and 2 = Low-watered.

Run 2 NW Smarthouse conveyor layout of Lines

Run 2 NW Positions

Lines	23	22	21	20	19	18	17	16	15	14	13	12	11	10	9	8	7	6	5	4	3	2
1	572	572	594	594	1	1	998	998	995	995	779	779	585	585	792	792	361	361	354	354	832	832
2	578	578	377	377	333	333	814	814	1019	1019	595	595	819	819	1	1	1	1	824	824	806	806
3	593	593	1017	1017	573	573	335	335	1	1	589	589	557	557	1012	1012	1	1	570	570	834	834
4	787	787	975	975	820	820	338	338	972	972	812	812	379	379	997	997	1	1	368	368	565	565
5	1006	1006	817	817	816	816	994	994	981	981	1	1	808	808	549	549	369	369	600	600	601	601
6	795	795	809	809	1	1	1	1	345	345	558	558	382	382	576	576	375	375	791	791	343	343
7	1	1	986	986	582	582	592	592	579	579	978	978	550	550	828	828	344	344	1016	1016	2	2
8	825	825	1	1	554	554	1	1	376	376	1013	1013	829	829	810	810	826	826	990	990	599	599
9	977	977	1	1	815	815	378	378	835	835	973	973	581	581	804	804	1014	1014	575	575	980	980
10	1	1	556	556	341	341	979	979	823	823	782	782	991	991	1022	1022	568	568	788	788	794	794
11	337	337	800	800	1009	1009	789	789	1007	1007	555	555	1	1	1	1	971	971	360	360	790	790
12	818	818	1	1	976	976	590	590	1	1	372	372	786	786	571	571	831	831	362	362	552	552
13	569	569	353	353	1	1	340	340	807	807	364	364	801	801	1	1	363	363	584	584	577	577
14	813	813	1003	1003	1	1	784	784	559	559	793	793	1024	1024	331	331	989	989	803	803	1	1
15	385	385	365	365	805	805	566	566	996	996	380	380	783	783	596	596	822	822	1	1	1004	1004
16	574	574	586	586	780	780	827	827	330	330	342	342	587	587	336	336	352	352	2	2	1	1
17	2	2	1026	1026	974	974	564	564	798	798	1	1	580	580	598	598	567	567	983	983	588	588
18	383	383	811	811	1021	1021	591	591	370	370	1	1	357	357	603	603	1011	1011	781	781	347	347
19	373	373	1	1	356	356	359	359	1000	1000	785	785	1	1	381	381	1001	1001	346	346	358	358
20	1	1	374	374	348	348	1023	1023	339	339	351	351	1	1	583	583	332	332	802	802	551	551
21	366	366	1002	1002	1005	1005	1	1	1015	1015	350	350	1008	1008	999	999	1	1	830	830	796	796
22	984	984	1010	1010	799	799	334	334	1	1	367	367	988	988	384	384	987	987	1	1	1018	1018
23	553	553	1020	1020	560	560	563	563	833	833	2	2	597	597	821	821	561	561	1	1	993	993
24	1025	1025	985	985	602	602	371	371	797	797	562	562	349	349	982	982	355	355	992	992	1	1

The correspondence between the numbers in this figure and the Lines is given in the Lines key sheet of the Excel spreadsheet. The replicated check line Navigator is coloured green, the recipient line Barke is grey and the unreplicated, offspring lines are blue.

Run 2 NW Smarthouse conveyor layout of Conditions

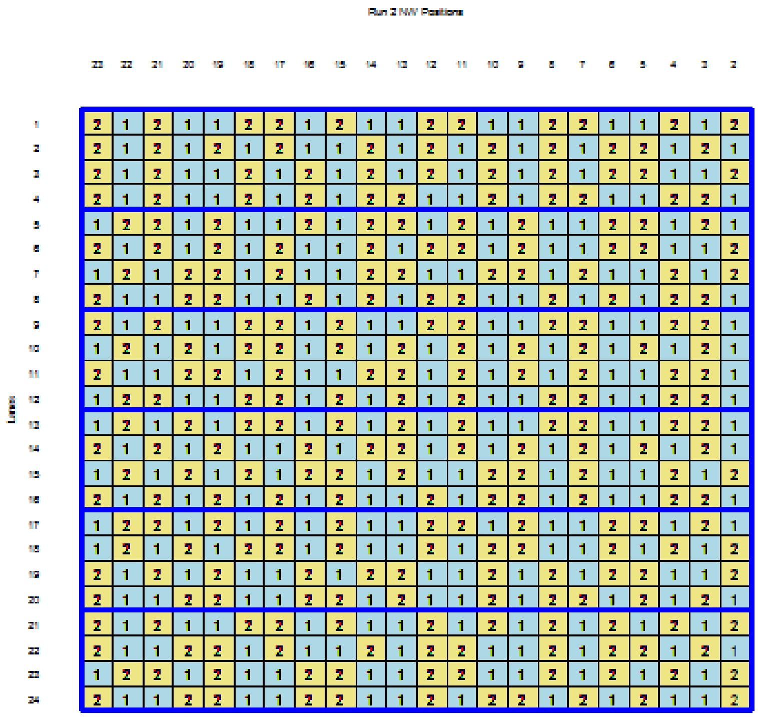

In this diagram, 1 = Well-watered and 2 = Low-watered.

Run 2 NE Smarthouse conveyor layout of Lines

Run 2 NE Positions

Lines

	2	3	4	5	6	7	8	9	10	11	12	13	14	15	16	17	18	19	20	21	22	23
1	238	238	1215	1215	271	271	101	101	1190	1190	226	226	866	866	1179	1179	269	269	1	1	1	1
2	72	72	241	241	838	838	248	248	59	59	1	1	1177	1177	257	257	1222	1222	68	68	1226	1226
3	856	856	874	874	867	867	1221	1221	1204	1204	2	2	1	1	1225	1225	231	231	236	236	883	883
4	1	1	268	268	1191	1191	1194	1194	235	235	843	843	844	844	1	1	96	96	81	81	849	849
5	214	214	66	66	242	242	88	88	1	1	836	836	75	75	1209	1209	98	98	232	232	1217	1217
6	71	71	233	233	229	229	74	74	215	215	79	79	1207	1207	1	1	853	853	1	1	1201	1201
7	862	862	1	1	261	261	2	2	239	239	1182	1182	1233	1233	1176	1176	1200	1200	225	225	1218	1218
8	62	62	871	871	1	1	272	272	877	877	83	83	99	99	237	237	1	1	1187	1187	869	869
9	1	1	1210	1210	84	84	70	70	260	260	227	227	240	240	1178	1178	1	1	892	892	851	851
10	882	882	868	868	1181	1181	1	1	852	852	253	253	2	2	884	884	95	95	85	85	78	78
11	247	247	857	857	104	104	216	216	1211	1211	859	859	1	1	251	251	1186	1186	842	842	93	93
12	254	254	878	878	1228	1228	243	243	1198	1198	1	1	1180	1180	1	1	267	267	219	219	222	222
13	1227	1227	266	266	1216	1216	259	259	1	1	64	64	1205	1205	69	69	90	90	224	224	1	1
14	92	92	234	234	1193	1193	263	263	256	256	1175	1175	76	76	890	890	245	245	1	1	60	60
15	262	262	1231	1231	1	1	855	855	1208	1208	1224	1224	1220	1220	94	94	102	102	1	1	228	228
16	244	244	885	885	252	252	86	86	73	73	887	887	864	864	87	87	1	1	91	91	875	875
17	77	77	1219	1219	841	841	1	1	1185	1185	80	80	213	213	65	65	82	82	61	61	1	1
18	1	1	860	860	1196	1196	858	858	1	1	845	845	1232	1232	854	854	1189	1189	223	223	1192	1192
19	891	891	1	1	1203	1203	270	270	265	265	97	97	879	879	872	872	1213	1213	67	67	889	889
20	1223	1223	2	2	1183	1183	886	886	103	103	1	1	863	863	249	249	1229	1229	837	837	105	105
21	264	264	220	220	258	258	1	1	1188	1188	1199	1199	221	221	881	881	873	873	230	230	246	246
22	1212	1212	1	1	89	89	1184	1184	255	255	847	847	250	250	1230	1230	1195	1195	848	848	218	218
23	861	861	1214	1214	888	888	840	840	876	876	1206	1206	1	1	1197	1197	1	1	839	839	106	106
24	1202	1202	880	880	1	1	100	100	1	1	217	217	63	63	870	870	846	846	865	865	850	850

The correspondence between the numbers in this figure and the Lines is given in the Lines key sheet of the Excel spreadsheet. The replicated check line Navigator is coloured green, the recipient line Barke is grey and the unreplicated, offspring lines are blue.

Run 2 NE Smarthouse conveyor layout of Conditions

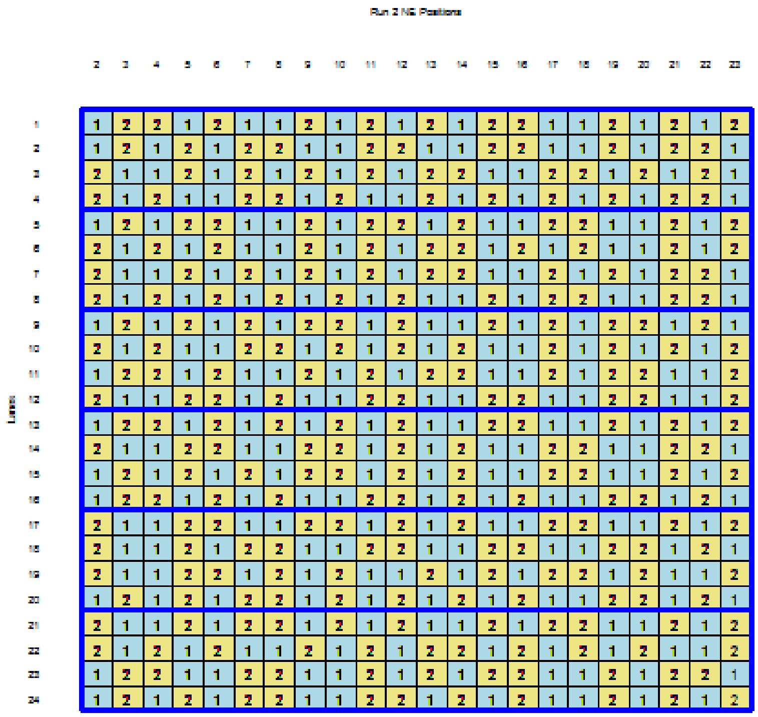

In this diagram, 1 = Well-watered and 2 = Low-watered.

Run 3 NW Smarthouse conveyor layout of Lines

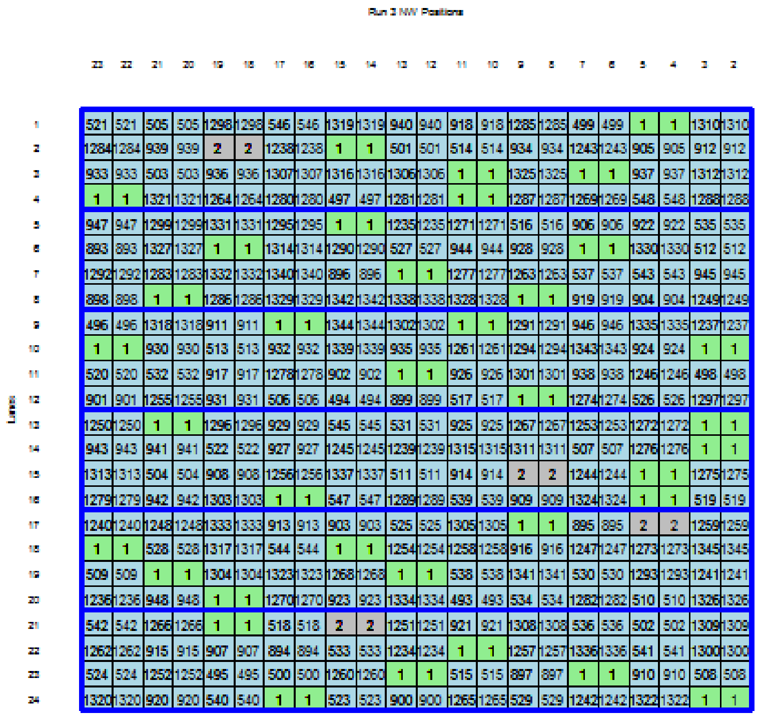

Run 3 NW Positions

Lines	23	22	21	20	19	18	17	16	15	14	13	12	11	10	9	8	7	6	5	4	3	2
1	521	521	505	505	1298	1298	546	546	1319	1319	940	940	918	918	1285	1285	499	499	1	1	1310	1310
2	1284	1284	939	939	2	2	1238	1238	1	1	501	501	514	514	934	934	1243	1243	905	905	912	912
3	933	933	503	503	936	936	1307	1307	1316	1316	1306	1306	1	1	1325	1325	1	1	937	937	1312	1312
4	1	1	1321	1321	1264	1264	1280	1280	497	497	1281	1281	1	1	1287	1287	1269	1269	548	548	1288	1288
5	947	947	1299	1299	1331	1331	1295	1295	1	1	1235	1235	1271	1271	516	516	906	906	922	922	535	535
6	893	893	1327	1327	1	1	1314	1314	1290	1290	527	527	944	944	928	928	1	1	1330	1330	512	512
7	1292	1292	1283	1283	1332	1332	1340	1340	896	896	1	1	1277	1277	1263	1263	537	537	543	543	945	945
8	898	898	1	1	1286	1286	1329	1329	1342	1342	1338	1338	1328	1328	1	1	919	919	904	904	1249	1249
9	496	496	1318	1318	911	911	1	1	1344	1344	1302	1302	1	1	1291	1291	946	946	1335	1335	1237	1237
10	1	1	930	930	513	513	932	932	1339	1339	935	935	1261	1261	1294	1294	1343	1343	924	924	1	1
11	520	520	532	532	917	917	1278	1278	902	902	1	1	926	926	1301	1301	938	938	1246	1246	498	498
12	901	901	1255	1255	931	931	506	506	494	494	899	899	517	517	1	1	1274	1274	526	526	1297	1297
13	1250	1250	1	1	1296	1296	929	929	545	545	531	531	925	925	1267	1267	1253	1253	1272	1272	1	1
14	943	943	941	941	522	522	927	927	1245	1245	1239	1239	1315	1315	1311	1311	507	507	1276	1276	1	1
15	1313	1313	504	504	908	908	1256	1256	1337	1337	511	511	914	914	2	2	1244	1244	1	1	1275	1275
16	1279	1279	942	942	1303	1303	1	1	547	547	1289	1289	539	539	909	909	1324	1324	1	1	519	519
17	1240	1240	1248	1248	1333	1333	913	913	903	903	525	525	1305	1305	1	1	895	895	2	2	1259	1259
18	1	1	528	528	1317	1317	544	544	1	1	1254	1254	1258	1258	916	916	1247	1247	1273	1273	1345	1345
19	509	509	1	1	1304	1304	1323	1323	1268	1268	1	1	538	538	1341	1341	530	530	1293	1293	1241	1241
20	1236	1236	948	948	1	1	1270	1270	923	923	1334	1334	493	493	534	534	1282	1282	510	510	1326	1326
21	542	542	1266	1266	1	1	518	518	2	2	1251	1251	921	921	1308	1308	536	536	502	502	1309	1309
22	1262	1262	915	915	907	907	894	894	533	533	1234	1234	1	1	1257	1257	1336	1336	541	541	1300	1300
23	524	524	1252	1252	495	495	500	500	1260	1260	1	1	515	515	897	897	1	1	910	910	508	508
24	1320	1320	920	920	540	540	1	1	523	523	900	900	1265	1265	529	529	1242	1242	1322	1322	1	1

The correspondence between the numbers in this figure and the Lines is given in the Lines key sheet of the Excel spreadsheet. The replicated check line Navigator is coloured green, the recipient line Barke is grey and the unreplicated, offspring lines are blue.

Run 3 NW Smarthouse conveyor layout of Conditions

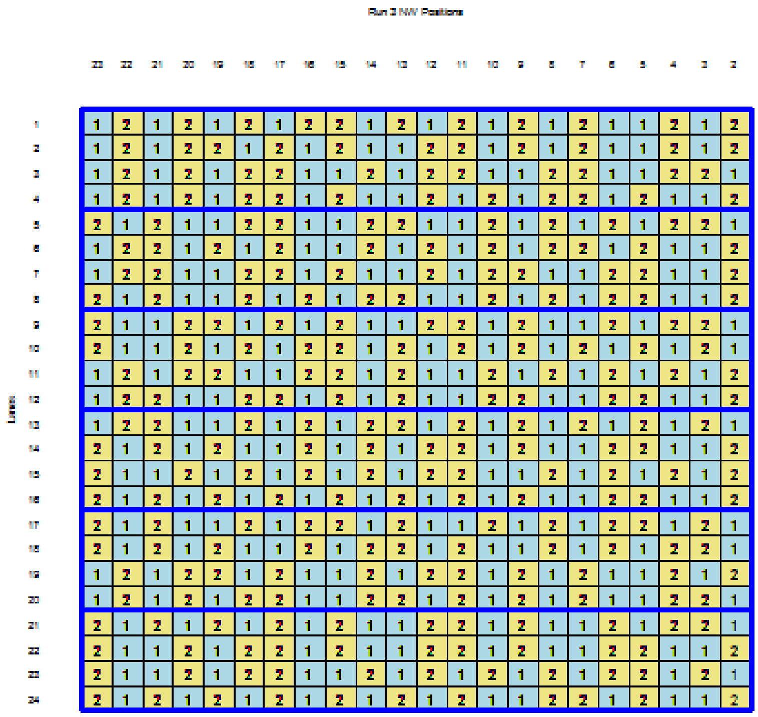

In this diagram, 1 = Well-watered and 2 = Low-watered.

Run 3 NE Smarthouse conveyor layout of Lines

Run 3 NE Positions

Lines	2	3	4	5	6	7	8	9	10	11	12	13	14	15	16	17	18	19	20	21	22	23
1	1	1	734	734	304	304	775	775	311	311	41	41	16	16	413	413	36	36	285	285	773	773
2	48	48	292	292	1	1	291	291	309	309	417	417	438	438	757	757	301	301	395	395	1	1
3	290	290	1	1	49	49	289	289	428	428	727	727	295	295	758	758	286	286	387	387	739	739
4	749	749	745	745	769	769	53	53	1	1	1	1	409	409	315	315	774	774	405	405	432	432
5	1	1	274	274	34	34	45	45	273	273	1	1	35	35	303	303	30	30	310	310	737	737
6	777	777	386	386	435	435	322	322	750	750	433	433	305	305	323	323	415	415	1	1	325	325
7	6	6	294	294	726	726	276	276	1	1	278	278	1	1	729	729	741	741	302	302	748	748
8	1	1	400	400	299	299	39	39	47	47	277	277	422	422	2	2	3	3	275	275	9	9
9	753	753	389	389	298	298	733	733	279	279	407	407	324	324	736	736	281	281	1	1	318	318
10	410	410	406	406	730	730	14	14	56	56	24	24	33	33	50	50	1	1	434	434	1	1
11	4	4	316	316	396	396	1	1	1	1	423	423	762	762	746	746	29	29	754	754	21	21
12	416	416	767	767	57	57	766	766	18	18	1	1	419	419	418	418	306	306	761	761	771	771
13	51	51	293	293	412	412	38	38	320	320	1	1	46	46	43	43	732	732	430	430	1	1
14	743	743	37	37	402	402	300	300	1	1	426	426	28	28	723	723	26	26	776	776	437	437
15	728	728	414	414	1	1	5	5	738	738	312	312	1	1	280	280	25	25	8	8	429	429
16	2	2	764	764	12	12	411	411	388	388	401	401	328	328	760	760	10	10	1	1	327	327
17	282	282	1	1	740	740	287	287	436	436	329	329	770	770	42	42	1	1	408	408	765	765
18	40	40	11	11	23	23	296	296	31	31	54	54	759	759	284	284	1	1	393	393	22	22
19	44	44	1	1	724	724	1	1	763	763	756	756	752	752	747	747	20	20	725	725	778	778
20	431	431	2	2	52	52	744	744	731	731	398	398	399	399	1	1	319	319	317	317	424	424
21	403	403	19	19	15	15	314	314	32	32	772	772	55	55	1	1	391	391	2	2	283	283
22	321	321	421	421	1	1	427	427	420	420	27	27	390	390	392	392	58	58	742	742	397	397
23	17	17	722	722	425	425	7	7	394	394	308	308	1	1	288	288	1	1	297	297	313	313
24	326	326	751	751	1	1	1	1	404	404	755	755	768	768	735	735	439	439	13	13	307	307

The correspondence between the numbers in this figure and the Lines is given in the Lines key sheet of the Excel spreadsheet. The replicated check line Navigator is coloured green, the recipient line Barke is grey and the unreplicated, offspring lines are blue.

Run 3 NE Smarthouse conveyor layout of Conditions

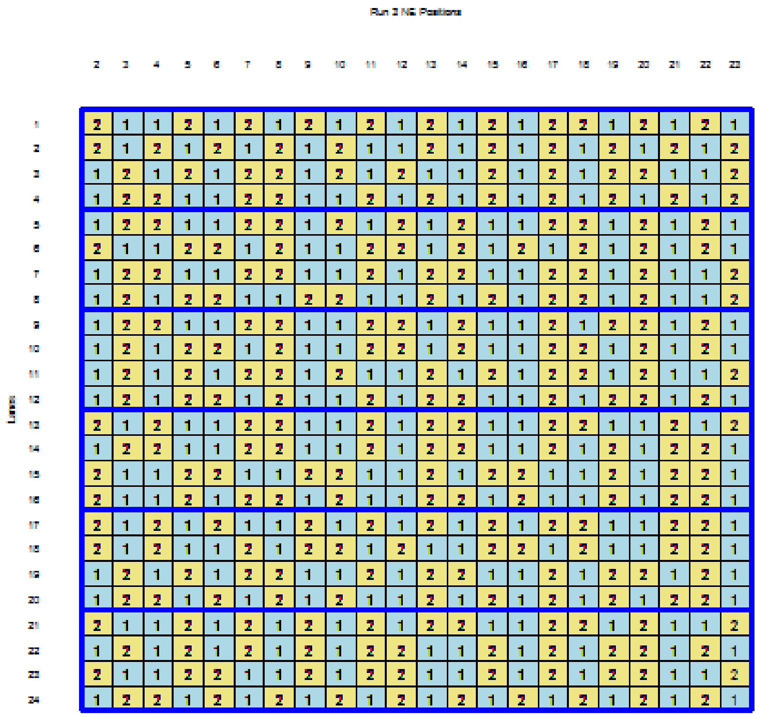

In this diagram, 1 = Well-watered and 2 = Low-watered.

W Smarthouse

West Smarthouse

Table 1

Position	1	2	3	4	5
2	Lane1	Lane2	Lane3	Lane4	Lane5
3	Lane1	Lane2	Lane3	Lane4	Lane5
4	Lane1	Lane2	Lane3	Lane4	Lane5
5	Lane1	Lane2	Lane3	Lane4	Lane5
6	Lane1	Lane2	Lane3	Lane4	Lane5
7	Lane1	Lane2	Lane3	Lane4	Lane5
8	Lane1	Lane2	Lane3	Lane4	Lane5
9	Lane1	Lane2	Lane3	Lane4	Lane5
10	Lane1	Lane2	Lane3	Lane4	Lane5
11	Lane1	Lane2	Lane3	Lane4	Lane5
12	Lane1	Lane2	Lane3	Lane4	Lane5
13	Lane1	Lane2	Lane3	Lane4	Lane5

Table 3

	1	2	3	4	5
1	Lane6	Lane7	Lane8	Lane9	Lane10
2	Lane6	Lane7	Lane8	Lane9	Lane10
3	Lane6	Lane7	Lane8	Lane9	Lane10
4	Lane6	Lane7	Lane8	Lane9	Lane10
5	Lane6	Lane7	Lane8	Lane9	Lane10
6	Lane6	Lane7	Lane8	Lane9	Lane10
7	Lane6	Lane7	Lane8	Lane9	Lane10
8	Lane6	Lane7	Lane8	Lane9	Lane10
9	Lane6	Lane7	Lane8	Lane9	Lane10
10	Lane6	Lane7	Lane8	Lane9	Lane10
11	Lane6	Lane7	Lane8	Lane9	Lane10
12	Lane6	Lane7	Lane8	Lane9	Lane10

Table 5

1	2	3	4	5
Lane11	Lane12	Lane13	Lane14	Lane15
Lane11	Lane12	Lane13	Lane14	Lane15
Lane11	Lane12	Lane13	Lane14	Lane15
Lane11	Lane12	Lane13	Lane14	Lane15
Lane11	Lane12	Lane13	Lane14	Lane15
Lane11	Lane12	Lane13	Lane14	Lane15
Lane11	Lane12	Lane13	Lane14	Lane15
Lane11	Lane12	Lane13	Lane14	Lane15
Lane11	Lane12	Lane13	Lane14	Lane15
Lane11	Lane12	Lane13	Lane14	Lane15
Lane11	Lane12	Lane13	Lane14	Lane15
Lane11	Lane12	Lane13	Lane14	Lane15

Table 7

1	2	3	4	5
Lane16	Lane17	Lane18	Lane19	Lane20
Lane16	Lane17	Lane18	Lane19	Lane20
Lane16	Lane17	Lane18	Lane19	Lane20
Lane16	Lane17	Lane18	Lane19	Lane20
Lane16	Lane17	Lane18	Lane19	Lane20
Lane16	Lane17	Lane18	Lane19	Lane20
Lane16	Lane17	Lane18	Lane19	Lane20
Lane16	Lane17	Lane18	Lane19	Lane20
Lane16	Lane17	Lane18	Lane19	Lane20
Lane16	Lane17	Lane18	Lane19	Lane20
Lane16	Lane17	Lane18	Lane19	Lane20
Lane16	Lane17	Lane18	Lane19	Lane20

Table 9

1	2	3	4	5
Lane21	Lane22	Lane23	Lane24	x
Lane21	Lane22	Lane23	Lane24	x
Lane21	Lane22	Lane23	Lane24	x
Lane21	Lane22	Lane23	Lane24	x
Lane21	Lane22	Lane23	Lane24	x
Lane21	Lane22	Lane23	Lane24	x
Lane21	Lane22	Lane23	Lane24	x
Lane21	Lane22	Lane23	Lane24	x
Lane21	Lane22	Lane23	Lane24	x
Lane21	Lane22	Lane23	Lane24	x
Lane21	Lane22	Lane23	Lane24	x
Lane21	Lane22	Lane23	Lane24	x

West

Table 2

14	Lane1	Lane2	Lane3	Lane4	Lane5
15	Lane1	Lane2	Lane3	Lane4	Lane5
16	Lane1	Lane2	Lane3	Lane4	Lane5
17	Lane1	Lane2	Lane3	Lane4	Lane5
18	Lane1	Lane2	Lane3	Lane4	Lane5
19	Lane1	Lane2	Lane3	Lane4	Lane5
20	Lane1	Lane2	Lane3	Lane4	Lane5
21	Lane1	Lane2	Lane3	Lane4	Lane5
22	Lane1	Lane2	Lane3	Lane4	Lane5
23	Lane1	Lane2	Lane3	Lane4	Lane5

Table 4

13	Lane6	Lane7	Lane8	Lane9	Lane10
14	Lane6	Lane7	Lane8	Lane9	Lane10
15	Lane6	Lane7	Lane8	Lane9	Lane10
16	Lane6	Lane7	Lane8	Lane9	Lane10
17	Lane6	Lane7	Lane8	Lane9	Lane10
18	Lane6	Lane7	Lane8	Lane9	Lane10
19	Lane6	Lane7	Lane8	Lane9	Lane10
20	Lane6	Lane7	Lane8	Lane9	Lane10
21	Lane6	Lane7	Lane8	Lane9	Lane10
22	Lane6	Lane7	Lane8	Lane9	Lane10

Table 6

Lane11	Lane12	Lane13	Lane14	Lane15
Lane11	Lane12	Lane13	Lane14	Lane15
Lane11	Lane12	Lane13	Lane14	Lane15
Lane11	Lane12	Lane13	Lane14	Lane15
Lane11	Lane12	Lane13	Lane14	Lane15
Lane11	Lane12	Lane13	Lane14	Lane15
Lane11	Lane12	Lane13	Lane14	Lane15
Lane11	Lane12	Lane13	Lane14	Lane15
Lane11	Lane12	Lane13	Lane14	Lane15
Lane11	Lane12	Lane13	Lane14	Lane15

Table 8

Lane16	Lane17	Lane18	Lane19	Lane20
Lane16	Lane17	Lane18	Lane19	Lane20
Lane16	Lane17	Lane18	Lane19	Lane20
Lane16	Lane17	Lane18	Lane19	Lane20
Lane16	Lane17	Lane18	Lane19	Lane20
Lane16	Lane17	Lane18	Lane19	Lane20
Lane16	Lane17	Lane18	Lane19	Lane20
Lane16	Lane17	Lane18	Lane19	Lane20
Lane16	Lane17	Lane18	Lane19	Lane20
Lane16	Lane17	Lane18	Lane19	Lane20

Table 10

Lane21	Lane22	Lane23	Lane24	x
Lane21	Lane22	Lane23	Lane24	x
Lane21	Lane22	Lane23	Lane24	x
Lane21	Lane22	Lane23	Lane24	x
Lane21	Lane22	Lane23	Lane24	x
Lane21	Lane22	Lane23	Lane24	x
Lane21	Lane22	Lane23	Lane24	x
Lane21	Lane22	Lane23	Lane24	x
Lane21	Lane22	Lane23	Lane24	x
Lane21	Lane22	Lane23	Lane24	x

E Smarthouse

West Smarthouse

Table 1

Position	1	2	3	4	5
2	Lane24	Lane23	Lane22	Lane21	Lane20
3	Lane24	Lane23	Lane22	Lane21	Lane20
4	Lane24	Lane23	Lane22	Lane21	Lane20
5	Lane24	Lane23	Lane22	Lane21	Lane20
6	Lane24	Lane23	Lane22	Lane21	Lane20
7	Lane24	Lane23	Lane22	Lane21	Lane20
8	Lane24	Lane23	Lane22	Lane21	Lane20
9	Lane24	Lane23	Lane22	Lane21	Lane20
10	Lane24	Lane23	Lane22	Lane21	Lane20
11	Lane24	Lane23	Lane22	Lane21	Lane20
12	Lane24	Lane23	Lane22	Lane21	Lane20
13	Lane24	Lane23	Lane22	Lane21	Lane20

Table 3

	1	2	3	4	5
1	Lane19	Lane18	Lane17	Lane16	Lane15
2	Lane19	Lane18	Lane17	Lane16	Lane15
3	Lane19	Lane18	Lane17	Lane16	Lane15
4	Lane19	Lane18	Lane17	Lane16	Lane15
5	Lane19	Lane18	Lane17	Lane16	Lane15
6	Lane19	Lane18	Lane17	Lane16	Lane15
7	Lane19	Lane18	Lane17	Lane16	Lane15
8	Lane19	Lane18	Lane17	Lane16	Lane15
9	Lane19	Lane18	Lane17	Lane16	Lane15
10	Lane19	Lane18	Lane17	Lane16	Lane15
11	Lane19	Lane18	Lane17	Lane16	Lane15
12	Lane19	Lane18	Lane17	Lane16	Lane15

Table 5

1	2	3	4	5
Lane14	Lane13	Lane12	Lane11	Lane10
Lane14	Lane13	Lane12	Lane11	Lane10
Lane14	Lane13	Lane12	Lane11	Lane10
Lane14	Lane13	Lane12	Lane11	Lane10
Lane14	Lane13	Lane12	Lane11	Lane10
Lane14	Lane13	Lane12	Lane11	Lane10
Lane14	Lane13	Lane12	Lane11	Lane10
Lane14	Lane13	Lane12	Lane11	Lane10
Lane14	Lane13	Lane12	Lane11	Lane10
Lane14	Lane13	Lane12	Lane11	Lane10
Lane14	Lane13	Lane12	Lane11	Lane10
Lane14	Lane13	Lane12	Lane11	Lane10

Table 7

1	2	3	4	5
Lane9	Lane8	Lane7	Lane6	Lane5
Lane9	Lane8	Lane7	Lane6	Lane5
Lane9	Lane8	Lane7	Lane6	Lane5
Lane9	Lane8	Lane7	Lane6	Lane5
Lane9	Lane8	Lane7	Lane6	Lane5
Lane9	Lane8	Lane7	Lane6	Lane5
Lane9	Lane8	Lane7	Lane6	Lane5
Lane9	Lane8	Lane7	Lane6	Lane5
Lane9	Lane8	Lane7	Lane6	Lane5
Lane9	Lane8	Lane7	Lane6	Lane5
Lane9	Lane8	Lane7	Lane6	Lane5
Lane9	Lane8	Lane7	Lane6	Lane5

Table 9

1	2	3	4	5
Lane4	Lane3	Lane2	Lane1	x
Lane4	Lane3	Lane2	Lane1	x
Lane4	Lane3	Lane2	Lane1	x
Lane4	Lane3	Lane2	Lane1	x
Lane4	Lane3	Lane2	Lane1	x
Lane4	Lane3	Lane2	Lane1	x
Lane4	Lane3	Lane2	Lane1	x
Lane4	Lane3	Lane2	Lane1	x
Lane4	Lane3	Lane2	Lane1	x
Lane4	Lane3	Lane2	Lane1	x
Lane4	Lane3	Lane2	Lane1	x
Lane4	Lane3	Lane2	Lane1	x

West

Table 2

14	Lane24	Lane23	Lane22	Lane21	Lane20
15	Lane24	Lane23	Lane22	Lane21	Lane20
16	Lane24	Lane23	Lane22	Lane21	Lane20
17	Lane24	Lane23	Lane22	Lane21	Lane20
18	Lane24	Lane23	Lane22	Lane21	Lane20
19	Lane24	Lane23	Lane22	Lane21	Lane20
20	Lane24	Lane23	Lane22	Lane21	Lane20
21	Lane24	Lane23	Lane22	Lane21	Lane20
22	Lane24	Lane23	Lane22	Lane21	Lane20
23	Lane24	Lane23	Lane22	Lane21	Lane20

Table 4

13	Lane19	Lane18	Lane17	Lane16	Lane15
14	Lane19	Lane18	Lane17	Lane16	Lane15
15	Lane19	Lane18	Lane17	Lane16	Lane15
16	Lane19	Lane18	Lane17	Lane16	Lane15
17	Lane19	Lane18	Lane17	Lane16	Lane15
18	Lane19	Lane18	Lane17	Lane16	Lane15
19	Lane19	Lane18	Lane17	Lane16	Lane15
20	Lane19	Lane18	Lane17	Lane16	Lane15
21	Lane19	Lane18	Lane17	Lane16	Lane15
22	Lane19	Lane18	Lane17	Lane16	Lane15

Table 6

Lane14	Lane13	Lane12	Lane11	Lane10
Lane14	Lane13	Lane12	Lane11	Lane10
Lane14	Lane13	Lane12	Lane11	Lane10
Lane14	Lane13	Lane12	Lane11	Lane10
Lane14	Lane13	Lane12	Lane11	Lane10
Lane14	Lane13	Lane12	Lane11	Lane10
Lane14	Lane13	Lane12	Lane11	Lane10
Lane14	Lane13	Lane12	Lane11	Lane10
Lane14	Lane13	Lane12	Lane11	Lane10
Lane14	Lane13	Lane12	Lane11	Lane10

Table 8

Lane9	Lane8	Lane7	Lane6	Lane5
Lane9	Lane8	Lane7	Lane6	Lane5
Lane9	Lane8	Lane7	Lane6	Lane5
Lane9	Lane8	Lane7	Lane6	Lane5
Lane9	Lane8	Lane7	Lane6	Lane5
Lane9	Lane8	Lane7	Lane6	Lane5
Lane9	Lane8	Lane7	Lane6	Lane5
Lane9	Lane8	Lane7	Lane6	Lane5
Lane9	Lane8	Lane7	Lane6	Lane5
Lane9	Lane8	Lane7	Lane6	Lane5

Table 10

Lane4	Lane3	Lane2	Lane1	x
Lane4	Lane3	Lane2	Lane1	x
Lane4	Lane3	Lane2	Lane1	x
Lane4	Lane3	Lane2	Lane1	x
Lane4	Lane3	Lane2	Lane1	x
Lane4	Lane3	Lane2	Lane1	x
Lane4	Lane3	Lane2	Lane1	x
Lane4	Lane3	Lane2	Lane1	x
Lane4	Lane3	Lane2	Lane1	x
Lane4	Lane3	Lane2	Lane1	x
